# A meta‐analysis of deep brain structural shape and asymmetry abnormalities in 2,833 individuals with schizophrenia compared with 3,929 healthy volunteers via the ENIGMA Consortium

**DOI:** 10.1002/hbm.25625

**Published:** 2021-09-08

**Authors:** Boris A. Gutman, Theo G.M. van Erp, Kathryn Alpert, Christopher R. K. Ching, Dmitry Isaev, Anjani Ragothaman, Neda Jahanshad, Arvin Saremi, Artemis Zavaliangos‐Petropulu, David C. Glahn, Li Shen, Shan Cong, Dag Alnæs, Ole Andreas Andreassen, Nhat Trung Doan, Lars T. Westlye, Peter Kochunov, Theodore D. Satterthwaite, Daniel H. Wolf, Alexander J. Huang, Charles Kessler, Andrea Weideman, Dana Nguyen, Bryon A. Mueller, Lawrence Faziola, Steven G. Potkin, Adrian Preda, Daniel H. Mathalon, Juan Bustillo, Vince Calhoun, Judith M. Ford, Esther Walton, Stefan Ehrlich, Giuseppe Ducci, Nerisa Banaj, Fabrizio Piras, Federica Piras, Gianfranco Spalletta, Erick J. Canales‐Rodríguez, Paola Fuentes‐Claramonte, Edith Pomarol‐Clotet, Joaquim Radua, Raymond Salvador, Salvador Sarró, Erin W. Dickie, Aristotle Voineskos, Diana Tordesillas‐Gutiérrez, Benedicto Crespo‐Facorro, Esther Setién‐Suero, Jacqueline Mayoral van Son, Stefan Borgwardt, Fabienne Schönborn‐Harrisberger, Derek Morris, Gary Donohoe, Laurena Holleran, Dara Cannon, Colm McDonald, Aiden Corvin, Michael Gill, Geraldo Busatto Filho, Pedro G. P. Rosa, Mauricio H. Serpa, Marcus V. Zanetti, Irina Lebedeva, Vasily Kaleda, Alexander Tomyshev, Tim Crow, Anthony James, Simon Cervenka, Carl M Sellgren, Helena Fatouros‐Bergman, Ingrid Agartz, Fleur Howells, Dan J. Stein, Henk Temmingh, Anne Uhlmann, Greig I. de Zubicaray, Katie L. McMahon, Margie Wright, Derin Cobia, John G. Csernansky, Paul M. Thompson, Jessica A. Turner, Lei Wang

**Affiliations:** ^1^ Department of Biomedical Engineering Illinois Institute of Technology Chicago Illinois USA; ^2^ Institute for Information Transmission Problems (Kharkevich Institute) Moscow Russia; ^3^ Clinical Translational Neuroscience Laboratory, Department of Psychiatry and Human Behavior University of California Irvine Irvine California USA; ^4^ Center for the Neurobiology of Learning and Memory University of California Irvine Irvine California USA; ^5^ Department of Psychiatry and Behavioral Sciences Northwestern University Feinberg School of Medicine Chicago Illinois USA; ^6^ Imaging Genetics Center, Mark & Mary Stevens Neuroimaging & Informatics Institute, Keck School of Medicine University of Southern California Los Angeles California USA; ^7^ Department of Biomedical Engineering Duke University Durham North Carolina USA; ^8^ Department of biomedical engineering Oregon Health and Science university Portland Oregon USA; ^9^ Department of Psychiatry Boston Children's Hospital and Harvard Medical School Boston Massachusetts USA; ^10^ Department of Biostatistics, Epidemiology and Informatics University of Pennsylvania Philadelphia Pennsylvania USA; ^11^ NORMENT, Division of Mental Health and Addiction Oslo University Hospital & Institute of Clinical Medicine, University of Oslo Oslo Norway; ^12^ Department of Psychology University of Oslo Oslo Norway; ^13^ Department of Psychiatry University of Maryland School of Medicine Baltimore Maryland USA; ^14^ Department of Psychiatry University of Pennsylvania Perelman School of Medicine Philadelphia Pennsylvania USA; ^15^ Department of Pediatrics University of California Irvine Irvine California USA; ^16^ Department of Psychiatry and Behavioral Sciences University of Minnesota Minneapolis Minnesota USA; ^17^ Department of Psychiatry and Human Behavior University of California Irvine Irvine California USA; ^18^ Department of Psychiatry and Weill Institute for Neurosciences University of California San Francisco San Francisco California USA; ^19^ Judith Ford Mental Health VA San Francisco Healthcare System San Francisco California USA; ^20^ Departments of Psychiatry & Neuroscience University of New Mexico Albuquerque New Mexico USA; ^21^ Tri‐institutional Center for Translational Research in Neuroimaging and Data Science (TReNDS) [Georgia State University, Georgia Institute of Technology] Emory University Atlanta Georgia USA; ^22^ Department of Electrical and Computer Engineering The University of New Mexico Albuquerque New Mexico USA; ^23^ Department of Psychiatry and Behavioral Sciences University of California San Francisco California USA; ^24^ Department of Psychology University of Bath Bath UK; ^25^ Division of Psychological & Social Medicine and Developmental Neurosciences Faculty of Medicine, TU‐Dresden Dresden Germany; ^26^ Mental Health Department ASL Roma1 DSM Rome Italy; ^27^ Laboratory of Neuropsychiatry IRCCS Santa Lucia Foundation Rome Italy; ^28^ Menninger Department of Psychiatry and Behavioral Sciences Baylor College of Medicine Houston Texas USA; ^29^ FIDMAG Germanes Hospitalàries Research Foundation CIBERSAM Barcelona Spain; ^30^ Institut d'Investigacions Biomdiques August Pi i Sunyer (IDIBAPS) Barcelona Spain; ^31^ Centre for Addiction and Mental Health (CAMH) Toronto Canada; ^32^ Department of Radiology, IDIVAL Marqués de Valdecilla University Hospital Santander Spain; ^33^ University Hospital Virgen del Rocio, IBiS University of Sevilla, CIBERSAM Sevilla Spain; ^34^ University Hospital Marqués de Valdecilla IDIVAL, CIBERSAM Santander Spain; ^35^ Department of Psychiatry University of Basel Basel Switzerland; ^36^ Department of Psychiatry and Psychotherapy University of Lübeck Lübeck Germany; ^37^ Centre for Neuroimaging and Cognitive Genomics, Discipline of Biochemistry National University of Ireland Galway Galway Ireland; ^38^ Centre for Neuroimaging and Cognitive Genomics, School of Psychology National University of Ireland Galway Galway Ireland; ^39^ Clinical Neuroimaging Laboratory, Centre for Neuroimaging and Cognitive Genomics National University of Ireland Galway Galway Ireland; ^40^ Neuropsychiatric Genetics Research Group, Department of Psychiatry Trinity College Dublin Dublin Ireland; ^41^ Trinity College Institute of Neuroscience Trinity College Dublin Dublin Ireland; ^42^ Laboratory of Psychiatric Neuroimaging (LIM‐21), Departamento e Instituto de Psiquiatria Hospital das Clinicas HCFMUSP, Faculdade de Medicina, Universidade de Sao Paulo Sao Paulo SP Brazil; ^43^ Hospital Sirio‐Libanes Sao Paulo SP Brazil; ^44^ Laboratory of Neuroimaging and Multimodal Analysis Mental Health Research Center Moscow Russia; ^45^ Department of Endogenous Mental Disorders Mental Health Research Center Moscow Russia; ^46^ Department of Psychiatry University of Oxford Oxford UK; ^47^ Centre for Psychiatry Reserach, Department of Clinical Neuroscience Karolinska Institutet, & Stockholm Health Care Services, Region Stockholm Stockholm Sweden; ^48^ Department of Physiology and Pharmacology Karolinska Institutet Stockholm Sweden; ^49^ Department of Psychiatry and Mental Health, Faculty of Health Sciences University of Cape Town Cape Town WC South Africa; ^50^ Neuroscience Institute University of Cape Town, Cape Town WC South Africa; ^51^ SA MRC Unit on Risk & Resilience in Mental Disorders University of Cape Town Cape Town WC South Africa; ^52^ Department of Child and Adolescent Psychiatry TU Dresden Germany; ^53^ School of Psychology, Faculty of Health Queensland University of Technology (QUT) Brisbane QLD Australia; ^54^ School of Clinical Sciences Queensland University of Technology (QUT) Brisbane QLD Australia; ^55^ Queensland Brain Institute University of Queensland Brisbane QLD Australia; ^56^ Department of Psychology and Neuroscience Center Brigham Young University Provo Utah USA; ^57^ Psychology & Neuroscience Georgia State University Atlanta Georgia USA; ^58^ Department of Psychiatry and Behavioral Health Ohio State University Wexner Medical Center Columbus Ohio USA

**Keywords:** schizophrenia, structure, subcortical shape

## Abstract

Schizophrenia is associated with widespread alterations in subcortical brain structure. While analytic methods have enabled more detailed morphometric characterization, findings are often equivocal. In this meta‐analysis, we employed the harmonized ENIGMA shape analysis protocols to collaboratively investigate subcortical brain structure shape differences between individuals with schizophrenia and healthy control participants. The study analyzed data from 2,833 individuals with schizophrenia and 3,929 healthy control participants contributed by 21 worldwide research groups participating in the ENIGMA Schizophrenia Working Group. Harmonized shape analysis protocols were applied to each site's data independently for bilateral hippocampus, amygdala, caudate, accumbens, putamen, pallidum, and thalamus obtained from T1‐weighted structural MRI scans. Mass univariate meta‐analyses revealed more‐concave‐than‐convex shape differences in the hippocampus, amygdala, accumbens, and thalamus in individuals with schizophrenia compared with control participants, more‐convex‐than‐concave shape differences in the putamen and pallidum, and both concave and convex shape differences in the caudate. Patterns of exaggerated asymmetry were observed across the hippocampus, amygdala, and thalamus in individuals with schizophrenia compared to control participants, while diminished asymmetry encompassed ventral striatum and ventral and dorsal thalamus. Our analyses also revealed that higher chlorpromazine dose equivalents and increased positive symptom levels were associated with patterns of contiguous convex shape differences across multiple subcortical structures. Findings from our shape meta‐analysis suggest that common neurobiological mechanisms may contribute to gray matter reduction across multiple subcortical regions, thus enhancing our understanding of the nature of network disorganization in schizophrenia.

## Graphical abstract

Our manuscript reports the first, large prospective subcortical shape meta‐analysis to date, comparing individuals with schizophrenia and healthy controls. Twenty‐one institutions worldwide contributed 24 cross‐sectional study samples to this study via the Enhancing Neuro Imaging Genetics Through Meta‐Analysis (ENIGMA) Consortium, Schizophrenia Working Group, totaling 2,833 individuals with schizophrenia and 3,929 healthy controls. The study utilized a prospective meta‐analysis approach with state‐of‐the‐art harmonized shape analysis protocols. We report robust patterns of subcortical shape and asymmetry differences, and associations with medication and symptoms that spanned multiple neighboring subcortical structures, which have not been previously reported in individual studies with consistency.

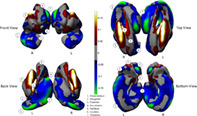

## 1 INTRODUCTION

Deep‐brain nuclei are implicated in a range of emotional, cognitive, motor, and sensory processes (Haijma et al., 2013; Levitt, Bobrow, Lucia, & Srinivasan, 2010; van Erp et al., 2016), with perturbations resulting in a host abnormal behavioral features (Bonelli & Cummings, 2007). Schizophrenia is associated with abnormalities in volume (Haijma et al., 2013; Levitt et al., 2010; van Erp et al., 2016) and shape measures (Csernansky et al., 1998; Gutman et al., 2015; Harms et al., 2007; Lee et al., 2004; Mamah et al., 2007; McClure et al., 2013; Narr et al., 2001; Roalf et al., 2015) of these subcortical structures, as well as their asymmetry (Okada et al., 2016; L. Wang, Joshi, Miller, & Csernansky, 2001). However, substantial heterogeneity in reported findings (see, e.g., Levitt et al., 2010 for a review) has inspired efforts to harmonize studies via collaborative meta‐analyses. Meta‐analyses aim to distill true effects from heterogeneous findings, a crucial task for advancing existing theories and for hypothesis generation (Berman & Parker, 2002). Prospective meta‐analyses allow for collaborative and harmonized analyses across diverse data sets (Jahanshad & Thompson, 2017; Thompson et al., 2014; van Erp et al., 2016). Using this approach, a recent meta‐analysis investigated subcortical volumes in over 4,500 individuals from 15 data sets, and found that schizophrenia patients had smaller hippocampus, amygdala, thalamus and nucleus accumbens, and larger pallidum as compared to healthy control participants, whereas caudate and putamen did not show group differences (van Erp et al., 2016).

Given the anatomical complexity of subcortical structures, studies of shape—defined as the inward and outward variations of surface‐based structural boundaries, as well as their curvatures, expansions, and contractions—can detect and characterize more nuanced disease‐related structural morphometry patterns relative to whole‐structure volume (Levitt et al., 2010). In particular, subcortical structural shape is consistently altered in subtle, yet significant, ways in individuals with schizophrenia (Csernansky, Wang, Joshi, Ratnanather, & Miller, 2004; Mamah, Alpert, Barch, Csernansky, & Wang, 2016; Yang et al., 2012). However, inconsistent findings for specific nuclei have been observed, likely due to the small sample sizes of individual studies as well as investigators' preferred choices of atlases, computational methods, statistical models, and specialized quantitative phenotypes.

For example, a review of shape analysis research in psychosis‐spectrum disorders reveals patient sample sizes and types ranging from 20 (Li et al., 2015) to 126 (Qiu et al., 2010) in “strict” schizophrenia samples, 28 (Qiu, Gan, Wang, & Sim, 2013) to 35 (Coscia et al., 2009) in first‐episode samples, 60 in an antipsychotic‐naïve schizophrenia sample, and another study of 86 early‐onset subjects (Chakravarty et al., 2015). While the choices for computational approaches in shape analysis are not as varied relative to volumetric procedures, there still exists some variability including: the radial distance method (Coscia et al., 2009; Thompson et al., 2003); FSL FIRST (Danivas, Kalmady, Venkatasubramanian, & Gangadhar, 2013; Li et al., 2015; Smith et al., 2004); large deformation diffeomorphic metric mapping (LDDMM; (Csernansky et al., 2004; Khan, Wang, & Beg, 2008; Qiu et al., 2010; Qiu et al., 2013; Qiu & Miller, 2008; Vaillant, Qiu, Glaunes, & Miller, 2007; Wang et al., 2008; Womer et al., 2014); and the MAGeT automatic segmentation method (Chakravarty et al., 2013) with a modified surface‐based approach (Chakravarty et al., 2015; Lerch et al., 2008). The heterogeneity in these approaches has yielded both consistent (e.g., thalamus: Coscia et al., 2009; Csernansky, Schindler, et al., 2004; Danivas et al., 2013; Harms et al., 2007; basal ganglia: Chakravarty et al., 2015; Mamah et al., 2016; Wang et al., 2008; hippocampus: Qiu et al., 2013; Wang et al., 2001) and inconsistent findings (e.g., Li et al., 2015), suggesting a need for synthesizing the literature to better capture the global effects of psychosis on these important brain regions.

Given the variability described above, a new approach to meta‐analysis of structural shape is needed to overcome the limitations of a traditional, retrospective meta‐analysis to characterize subcortical structural shape alterations in schizophrenia. The ENIGMA consortium (Thompson et al., 2020) recently developed a prospective shape meta‐analysis approach by harmonizing atlases, computational methods, and statistical models prior to hypothesis testing (Gutman et al., 2015; Gutman, Wang, Rajagopalan, Toga, & Thompson, 2012; Roshchupkin et al., 2016). Here we report the first, large prospective subcortical shape meta‐analysis comparing individuals with schizophrenia and healthy control participants using standardized normalization to a common surface‐based subcortical atlas, harmonized quality assurance procedures, and a common set of linear statistical models. In this approach, shape analysis was first independently performed for each data set, followed by a meta‐analysis on the aggregated group‐level results. Two measures of shape were computed; the first was a measure of distance from the medial axis of each structure which we called *thickness*, while the second was a measure of surface *contraction* or *expansion* as calculated by the log Jacobian determinant.

Based on previous literature findings, we hypothesized a predominance of concordant reductions in thickness and contractions across the surfaces of the hippocampus, amygdala, nucleus accumbens, and thalamus. Furthermore, we hypothesized a predominance of concordant increases in thickness and expansions across the surfaces of the putamen and pallidum, in individuals with schizophrenia as compared with control participants. Since atypical antipsychotic drugs have been associated with both increases (Haijma et al., 2013; Konradi & Heckers, 2001; Roiz‐Santianez, Suarez‐Pinilla, & Crespo‐Facorro, 2015) and reductions (Dorph‐Petersen et al., 2005; Ho, Andreasen, Ziebell, Pierson, & Magnotta, 2011; Li et al., 2018; Roiz‐Santianez et al., 2014; Roiz‐Santianez et al., 2015) in subcortical volumes, as well as shape alterations (Mamah et al., 2012), we also attempted to resolve these disparate findings by testing for effects of antipsychotic drug exposure (using chlorpromazine dose equivalents) on shape.

## 2 MATERIALS AND METHODS

Twenty‐four worldwide cross‐sectional study samples from 21 institutions (Figure S1)—2,833 individuals with schizophrenia and 3,929 healthy control participants—contributed to the collaborative shape meta‐analysis via the ENIGMA Schizophrenia Working Group. Each study sample was collected with participants' written informed consent approved by local Institutional Review Boards. The characteristics of these cohorts are provided in Table 1 including weighted age, age of illness onset, duration of illness, and symptom severity. The weighted total scores for positive and negative syndrome scale (PANSS), scale for the assessment of negative symptoms (SANS), and scale for the assessment of positive symptoms (SAPS) across data sets were 16.6 (range of means across data sets: 13.7–22.9), 21.3 (range: 5.5–38.9) and 17.1 (range: 9.0–23.2), respectively. For data sets that recorded current antipsychotic type and dose, we determined the percentage of patients on second‐generation (atypical; 77%), first‐generation (typical; 15%), both (3%) or none (5%), and chlorpromazine dose equivalents (from 11 data sets) based on Woods (2003) (http://www.scottwilliamwoods.com/files/Equivtext.doc).

**TABLE 1:** Sample characteristics

Mean (range of means)	Schizophrenia	Healthy control
*N*	2,833	3,929
% male	66.9% (54.4–100%)	54.9% (38.6–100%)
Age, years	35.1 (16.3–43.9)	33.2 (16.2–43.6)
Age at onset, years	23.9 (20.7–28.9)	‐
Duration of illness, years	11.3 (0.7–31.5)	‐
PANSS total	16.6 (13.7–22.9)	‐
SANS total	21.3 (5.5–38.9)	‐
SAPS total	17.1 (9.0–23.2)	‐
Chlorpromazine dose equivalent	376 (166.1–634.6)	‐

*Note*: Age, age at onset and duration of illness, total PANSS (Positive and Negative Syndrome Scale), SANS (Scale for the Assessment of Negative Symptoms), SAPS (Scale for the Assessment of Positive Symptoms), and chlorpromazine dose equivalents were all weighted by sample size. Range is reported on range of means across the data sets. The 100% extreme in % male was due to the all‐male sample from the Mental Health Research Center, Moscow.

To assess subcortical shape measures, we followed the validated ENIGMA‐Shape pipeline (http://enigma.usc.edu/ongoing/enigma-shape-analysis/) (Ching et al., 2020; Chye et al., 2020; T. C. Ho et al., 2020; Roshchupkin et al., 2016). A set of standardized scripts to compute mass univariate statistics was distributed to all sites performing the analysis locally via the ENIGMA‐Git webpage (https://github.com/ENIGMA-git/ENIGMA/tree/master/WorkingGroups). To harmonize analysis across sites, the distributed script accessed a static online list of models agreed upon by the ENIGMA‐Schizophrenia Working Group. Below, we summarize the pipeline.

1. FreeSurfer parcellation. Left and right thalamus, caudate, putamen, pallidum, accumbens, hippocampus and amygdala parcellations, their volumes, and intracranial volumes (ICVs), were obtained with FreeSurfer (http://surfer.nmr.mgh.harvard.edu) from high‐resolution T1‐weighted structural brain scans. For details on study type (single site or multisite), scanner vendor/strength/sequence, acquisition parameters, FreeSurfer versions, and parcellation quality control, we refer the reader to van Erp et al. (2016) table S3b.
2. Surface triangulation. The boundary of each individual FreeSurfer‐parcellated subcortical region was represented by a triangulated surface model. The surface was spherically inflated and registered to a region‐specific average model by matching local geometric features (Wang et al., 2011). In this way, each subject was represented by a set of shapes with vertex‐to‐vertex correspondence to a standard template surface. Visual quality control of the surfaces was performed by individual raters according to the ENIGMA‐Shape Quality Control guide (http://enigma.ini.usc.edu/ongoing/enigma-shape-analysis/). Beyond the guide, sites performed local quality control, assisted by experienced raters at the University of Southern California Imaging Genetics Center for particularly difficult cases. More information on shape quality control and failure rates is available in Supplementary Materials.
3. Surface registration. A single template—the default ENIGMA shape atlas—was used by all ENIGMA‐Schizophrenia sites participating in the shape project. The ENIGMA‐Shape atlas was constructed by averaging surface models of 200 unrelated individuals (100 M/100 F, mean age: 22.9 [2.8] years) from the Queensland Twin Imaging Study (QTIM, Renteria et al., 2014). QTIM data were not used in this study beyond atlas construction.
4. Shape and asymmetry computation. Following registration, a medial curve was computed for the individual shape models (Gutman et al., 2012), and their registrations were further refined (Gutman et al., 2012). Two measures of shape were calculated at each vertex (Wang et al., 2011). The first, which we called “thickness,” was the distance to the medial curve. For a cylindrical structure, the medial curve is its center axis, and our measure of thickness measures the length of the radius of each circular cross‐section. The second quantity was the Jacobian determinant, which we called “contraction or expansion,” was defined as the ratio of the triangular area relative to the triangular area in the template at corresponding vertices. As the Jacobian tends to be non‐Gaussian, we used its logarithm transform in all analyses. Vertex‐wise maps were then compared to test for group differences (see below). We reasoned that when significant thinning and contraction (or thickening and expansion) occurred concordantly in the same vertices, subvolume changes were likely occurring nearer the structural surface. In contrast, when measures of thickness and contraction/expansion were present in a discordant fashion, we reasoned that subvolume changes could be occurring farther from the surface and closer to the medial curve. We computed the degree of concordance for each of the structures. Finally, the ENIGMA‐Shape atlas contains corresponding vertices across the left and right hemispheres allowing calculation of asymmetry indices for the absolute difference between left and right thickness and the log Jacobian determinant at each vertex.
5. Statistical analysis. Vertex‐wise mass univariate analysis per shape/asymmetry measure was independently performed first for each data set. Resulting group‐level maps of effect sizes (Cohen's *d*; Cohen, 1992), regression parameters, and confidence intervals were then aggregated for mass univariate meta‐analysis. We followed the same meta‐analytic strategy as in van Erp et al. (2016), using inverse variance‐based mixed effects sample weighting with as implemented in the R *metafor* package. Further details are available in Supplementary Materials. The following effects were tested using linear models: effect of diagnosis on thickness (Model 1), log Jacobian determinant (Model 2), asymmetry index of thickness (Model 3), asymmetry index of log Jacobian determinant (Model 4), and effect of chlorpromazine dose equivalents in individuals with schizophrenia (Model 5). In all models, sex and age were accounted for by including their linear, quadratic, and interaction terms: sex, age, age x sex, age^2^, and age^2^ x sex. Intracranial Volume (ICV) was also included in all models as a covariate. Maps of *p*‐values resulting from the meta‐analysis were corrected for multiple comparisons (number of structures, vertices, and measures) using a modified searchlight false discovery rate (FDR) procedure (Kriegeskorte, Goebel, & Bandettini, 2006; Langers, Jansen, & Backes, 2007). The correction was applied globally across all structures and measures for each linear model. Distance in the searchlight procedure was defined as the Euclidean distance between atlas vertices, with distance between vertices of different structures set to infinity. We note that this is a more conservative correction than one that assumes spatial correlation between boundaries of different structures, though less conservative than the original Benjamini and Hochberg procedure (Hochberg & Benjamini, 1990).

## 3 RESULTS

### 3.1 Effect of diagnosis on bilateral shape features

**FIGURE 1:**
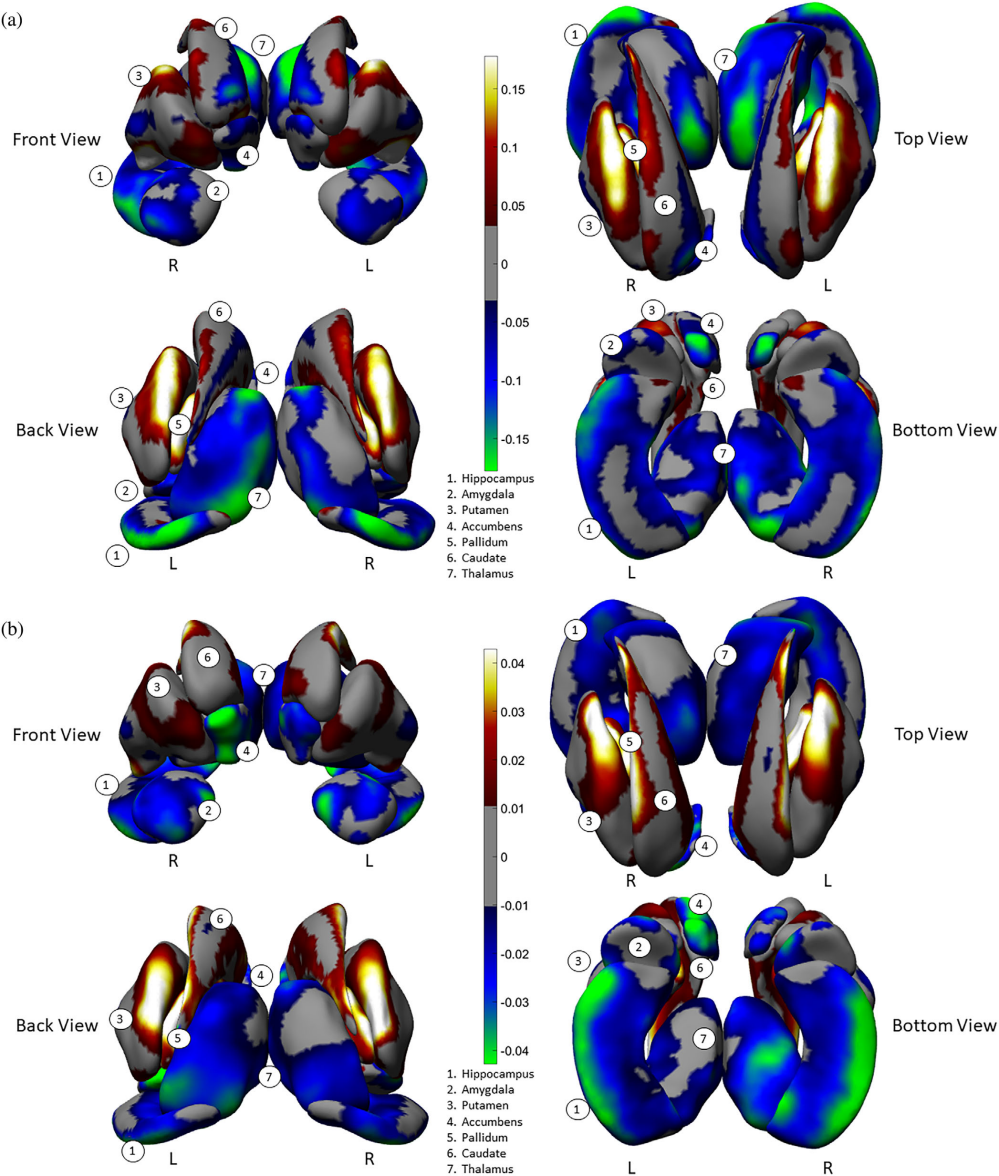
Vertex‐wise effects of diagnosis (i.e., schizophrenia vs. control) on each hemisphere for (a) thickness, (b) surface dilation/contraction (log Jacobian determinant). The effects are tested in models that included diagnosis sex, age, age x sex, age^2^, age^2^ x sex, and ICV. Vertex‐wise effect sizes (Cohen's *d*, see text) are visualized on subcortical surfaces. The subcortical structures—1. hippocampus, 2. amygdala, 3. putamen, 4. accumbens, 5. pallidum, 6. caudate, and 7. thalamus—are shown as a group situated in template space, from front, back, top, and bottom viewpoints of the brain. L = left hemisphere. R = right hemisphere. Color scale indicates the intensity of effect sizes. Cooler colors (i.e., negative effect sizes) indicate thinning, surface contraction for schizophrenia as compared to controls, and warmer colors (i.e., positive effect sizes) indicate thickening, surface dilation. Gray color indicates nonsignificant surface vertices after multiple comparison correction

Patterns of predominant concave shape differences were observed across multiple, neighboring subcortical structures. An examination of the aggregate surfaces of the hippocampus, amygdala, accumbens, and the thalamus indicate a predominance of regions showing thinning (Cohen's *d* range = −.03 to −.17; Figure 1a) and surface contractions (Cohen's *d* range = −.01 to −.04; Figure 1b) in individuals with schizophrenia as compared with control subjects. In contrast, an examination of the aggregate surfaces of the caudate, putamen and the pallidum, indicate a predominance of regions showing thickening (Cohen's *d* range = .03 to .17; Figure 1a) and surface expansions (Cohen's *d* range = .01 to .04; Figure 1b). The magnitude of size for all above comparisons are characterized as small effects (Cohen, 1992).

Group differences in the two shape measures did not always overlap with each other. For example, surface contraction without thinning was observed in the superior portions of the hippocampal body (i.e., close to the CA2‐4 + DG subfields, Figure S2). In addition, the caudate showed thinning along with surface expansion in its medial regions (Figure S7A,B). Nonetheless, the proportion of concordance of these two measures accounted for the majority of shape differences. A more detailed description of the spatial patterns and the degree of concordance of shape measures for each individual structure can be found in the Supplementary Materials.

**FIGURE 2:**
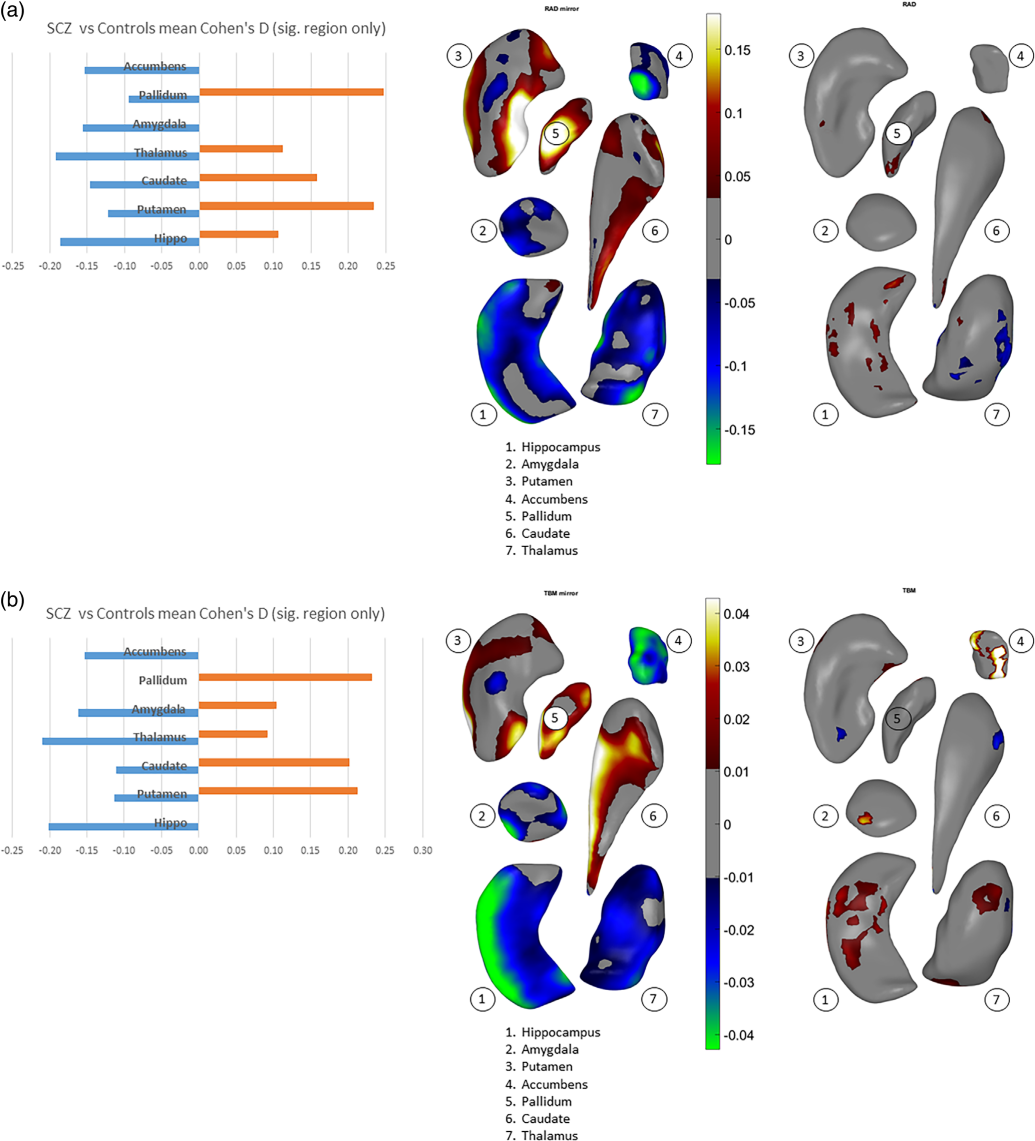
Overall and vertex‐wise effects of diagnosis (i.e., schizophrenia vs. control) across hemispheres for (a) thickness, (b) surface dilation/contraction (log Jacobian determinant). The effects are tested in models that included diagnosis sex, age, age x sex, age^2^, age^2^ x sex, and ICV. In the left column, mean positive effect sizes and mean negative effect sizes across each subcortical structure surface (see text) are shown as bar plots. The middle column shows the vertex‐wise effects of diagnosis on interhemispheric means (see text). The right column shows the vertex‐wise effects of diagnosis on interhemispheric absolute differences (reproduced from Figure 3a,b right columns). The subcortical structures—1. hippocampus, 2. amygdala, 3. putamen, 4. accumbens, 5. pallidum, 6. caudate, and 7. thalamus—are positioned generally from a bottom viewpoint, with some slightly rotated about their own principal axis to be oblique, for better exposure: caudate—pi/7 or about 25°, accumbens—pi/10 or 18°, pallidum—pi/3 or 60°. Color scale indicates the intensity of effect sizes. Cooler colors (i.e., negative effect sizes) indicate reduced asymmetry for schizophrenia as compared to controls, and warmer colors (i.e., positive effect sizes) indicate exaggerated asymmetry. Gray color indicates nonsignificant surface vertices after multiple comparison correction

We assessed an overall effect size for thinning/thickening and surface contraction/expansion for each subcortical structure collapsed across the hemispheres. We did this by first obtaining surface indices for which the above linear models were significant on measures of interhemispheric means, then separately computing an average for vertices carrying positive effect sizes and an average for negative effect sizes (see Cohen's *d*, Figure 2a,b). With regard to thinning vs. thickening (Figure 2a), only the amygdala and accumbens showed thinning (i.e., negative effect sizes only) in schizophrenia participants. For the hippocampus and thalamus, surface regions with thinning were larger in extent (based on effect size) compared with regions showing thickening. The caudate, putamen, and pallidum showed more thickening than thinning. With regard to surface contraction vs. expansion (Figure 2b), only the hippocampus and accumbens showed surface contraction in schizophrenia participants. Surface regions with larger contraction relative to expansion (based on effect size) included the amygdala and thalamus. The caudate and putamen showed more expansion than contraction, and the pallidum showed only expansion compared to control participants. See Supplementary Materials for more detailed descriptions. Again, the magnitude of all effects above were characterized as small in size.

### 3.2 Effect of diagnosis on shape asymmetry

**FIGURE 3:**
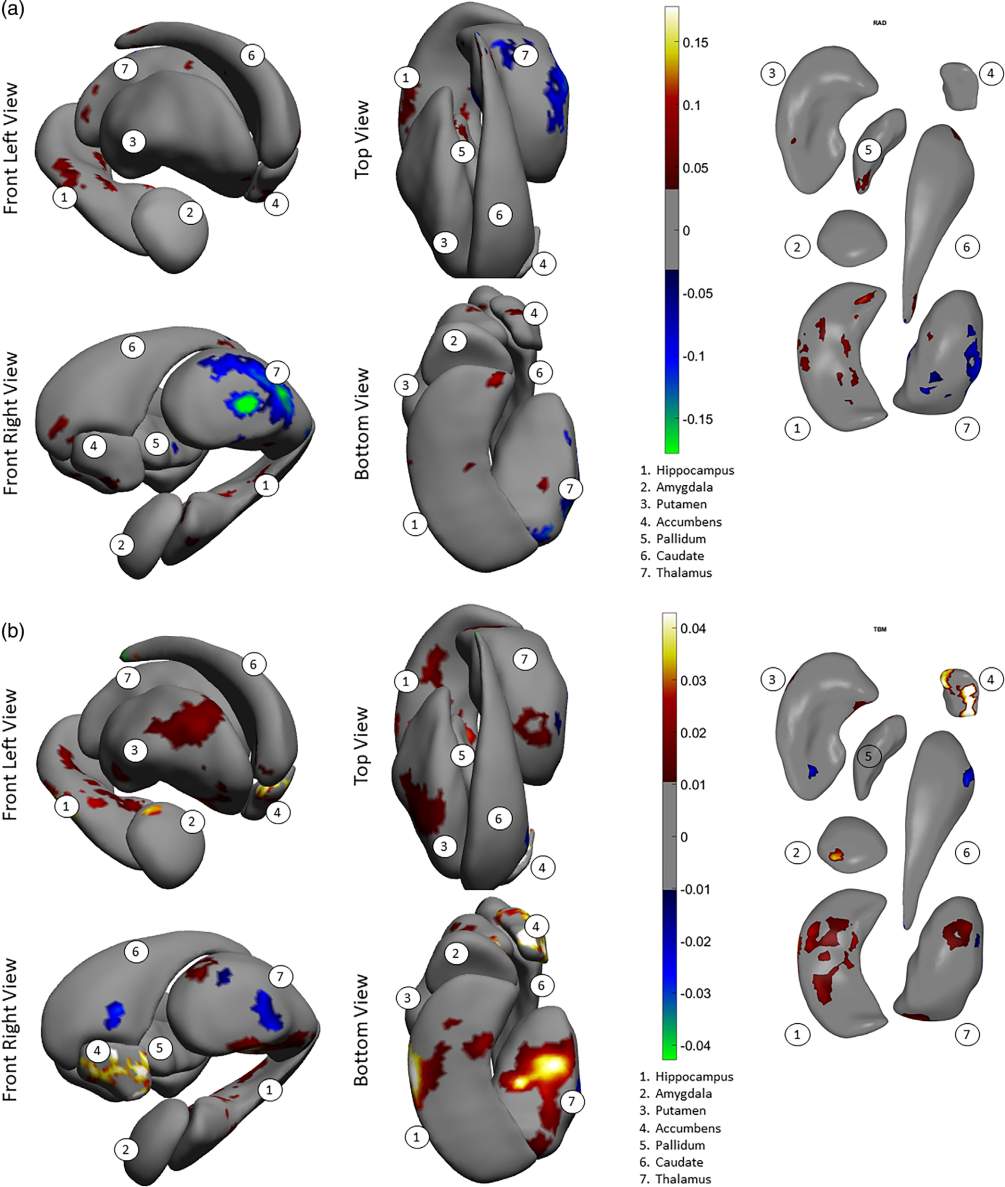
Effects of diagnosis (i.e., schizophrenia vs. control) for (a) asymmetry index of thickness, (b) asymmetry index of surface dilation/contraction (log Jacobian determinant). Vertex‐wise asymmetry indices for thickness and surface dilation/contraction were calculated as the absolute values of left‐versus‐right differences. The effects are tested in models that included diagnosis sex, age, age x sex, age^2^, age^2^ x sex, and ICV. Effect sizes (Cohen's *d*, see text) are visualized on subcortical surfaces. In the left two columns, the subcortical structures—1. hippocampus, 2. amygdala, 3. putamen, 4. accumbens, 5. pallidum, 6. caudate, and 7. thalamus—are shown as a group situated in template space, from front left, front right, top and bottom viewpoints of the brain. L = left hemisphere. R = right hemisphere. In the right column, the subcortical structures are positioned generally from a bottom viewpoint, with some slightly rotated about their own principal axis to be oblique, for better exposure: caudate—pi/7 or about 25°, accumbens—pi/10 or 18°, pallidum—pi/3 or 60°. Color scale indicates intensity of effect sizes. Cooler colors (i.e., negative effect sizes) indicate reduced asymmetry for schizophrenia as compared with controls, and warmer colors (i.e., positive effect sizes) indicate exaggerated asymmetry. Gray color indicates nonsignificant surface vertices after multiple comparison correction

Significant group differences in asymmetry patterns were also observed for both shape measures. Our interpretation of shape asymmetry was based on how the asymmetry index was calculated, which was the absolute difference in effect size for the structures between left and right hemispheres. The asymmetry index for thinning/thickening (Figure 3a) was greater for individuals with schizophrenia as compared to control subjects for the hippocampus, amygdala, nucleus accumbens, and putamen (see *red color* on the surfaces, Cohen's *d* = .05 to .1). For the thalamus, the index was predominantly smaller, as seen in *blue color* (Cohen's *d* = −0.05 to −0.15). For the pallidum and the caudate nucleus, group differences in the asymmetry index was negligible. The asymmetry index of surface contraction/expansion (Figure 3b) was greater for individuals with schizophrenia as compared with control subjects for the hippocampus, amygdala, putamen, and nucleus accumbens (Cohen's *d* = .01 to .04). For the thalamus, the index was predominantly greater for individuals with schizophrenia as compared with control subjects, with smaller proportions of the surface showing smaller indices (Cohen's *d* = −.01 to −.03). For the pallidum and caudate nucleus, group differences in the asymmetry index was negligible.

Consistent with the shape difference maps, patterns of shape asymmetry differences also demonstrated continuity across multiple, neighboring structures. For example, one pattern extended across the hippocampus, amygdala, to the thalamus, showing predominantly exaggerated asymmetry for individuals with schizophrenia as compared with control subjects (Figure 3b, *bottom and front views*), and another encompassed the ventral striatum and ventral and dorsal thalamus, revealing predominantly diminished normal asymmetry for individuals with schizophrenia (Figure 3b, *front view*). Additionally, the determination that these asymmetry differences indicated a leftward versus rightward, or an exaggerated versus diminished normal asymmetry, was made in the context of group differences across both hemispheres; please see Supplementary Materials for these observations.

### 3.3 Clinical correlates

**FIGURE 4:**
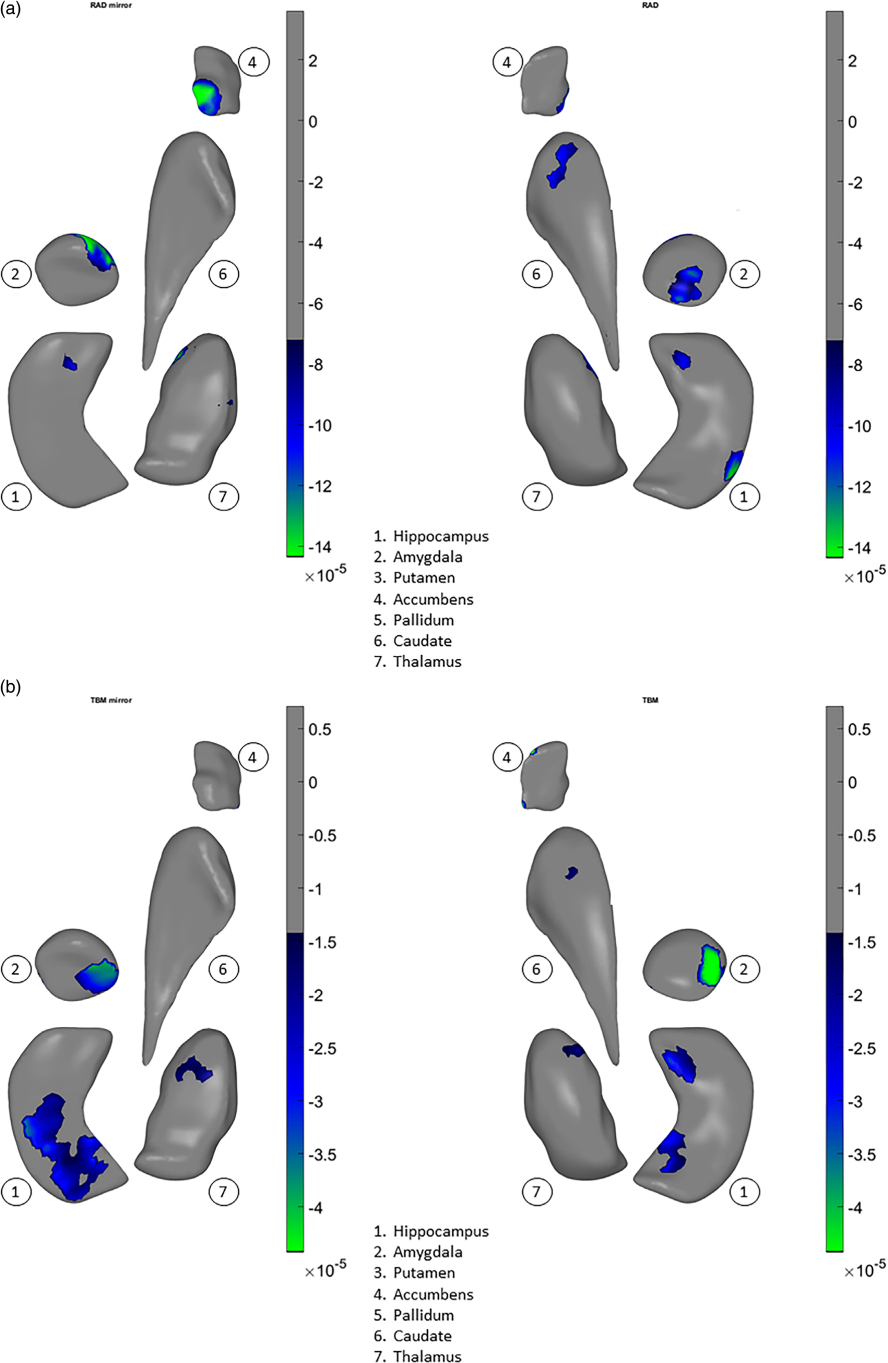
Overall and vertex‐wise effects of chlorpromazine dose equivalents across both hemispheres for (a) thickness, (b) surface dilation/contraction (log Jacobian determinant). The effects are tested in models that included diagnosis sex, age, age x sex, age^2^, age^2^ x sex, and ICV. Small but statistically significant relationships are found between higher chlorpromazine dose equivalents and locally reduced thickness (a) and surface contraction (b) in the hippocampus, amygdala, caudate, accumbens, and thalamus. In the left column, the subcortical structures—1. hippocampus, 2. amygdala, 4. accumbens, 6. caudate, and 7. thalamus—are positioned generally from a bottom viewpoint, with some slightly rotated about their own principal axis to be oblique, for better exposure: caudate—pi/7 or about 25°, accumbens—pi/10 or 18°. In the right column, the subcortical structures are positioned generally from a top viewpoint with the same rotations. Color scale indicates intensity of effect sizes. Cooler colors indicate negative associations, that is, higher chlorpromazine dose equivalents are associated with reduced surface measures. Gray color indicates nonsignificant surface vertices after multiple comparison correction. Putamen and pallidum are not shown as no effects were found for these subcortical structures

**FIGURE 5:**
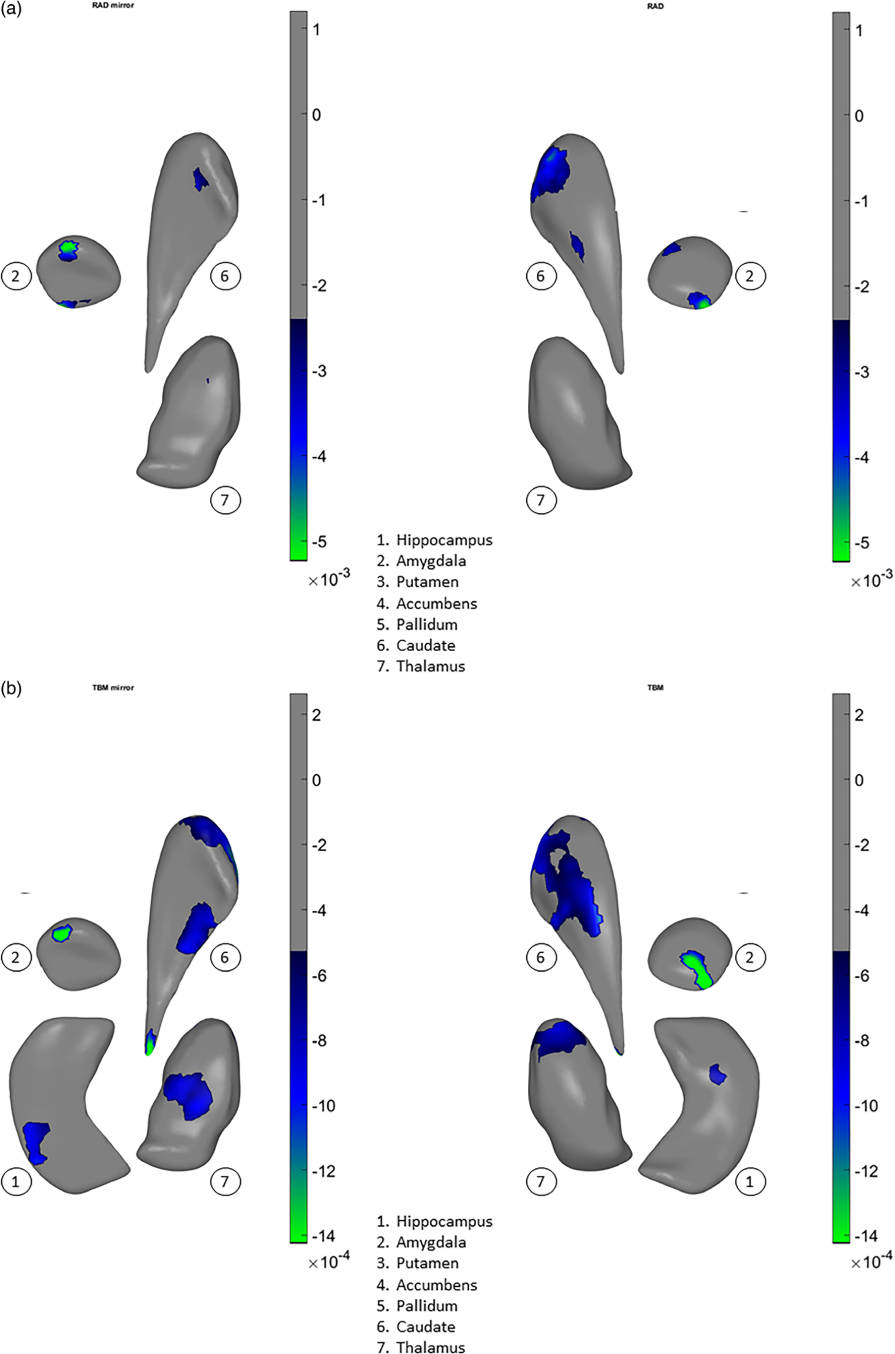
Overall and vertex‐wise effects of SAPS total scores across both hemispheres for (a) thickness, (b) surface dilation/contraction (log Jacobian determinant). The effects are tested in models that included diagnosis sex, age, age x sex, age^2^, age^2^ x sex, and ICV. Small but statistically significant relationships are found between higher SAPS total scores and locally reduced thickness in the amygdala, caudate, and thalamus (a), and surface contraction (b) in the hippocampus, amygdala, caudate, and thalamus. In the left column, the subcortical structures—1. hippocampus (panel b, surface contraction only), 2. amygdala, 6. caudate, and 7. thalamus—are positioned generally from a bottom viewpoint, with some slightly rotated about their own principal axis to be oblique, for better exposure: caudate—pi/7 or about 25°, accumbens—pi/10 or 18°. In the right column, the subcortical structures are positioned generally from a top viewpoint with the same rotations. Color scale indicates the intensity of effect sizes. Cooler colors indicate negative associations, that is, higher chlorpromazine dose equivalents are associated with reduced surface measures. Gray color indicates nonsignificant surface vertices after multiple comparison correction. Accumbens, putamen, and pallidum are not shown as no effects were found for these subcortical structures

In a prior study of structural volumes, we did not detect any significant correlations between chlorpromazine dose equivalents or psychopathology measures and volume differences in a large subset of the patients from 15 of 21 samples analyzed here (van Erp et al., 2016). However, in the present study, using our shape meta‐analysis, we found small but statistically significant relationships between higher chlorpromazine dose equivalents and shape‐derived thinning (Cohen's *d* = −.00008 to −.00014, Figure 4a) and surface contraction (Cohen's *d* = −.00005 to −.00004, Figure 4b) in the hippocampus and amygdala, and also in the caudate, accumbens, and thalamus. In addition, we found small but statistically significant relationships between elevated positive symptom (SAPS total) scores and thinning (Cohen's *d* = −.00002.5 to −.00005, Figure 5a) and contraction (Cohen's *d* = −.00005 to −.00014, Figure 5b) in the hippocampus, amygdala, caudate, and thalamus.

## 4 DISCUSSION

In this meta‐analysis of subcortical shape variations in schizophrenia, we found a predominance of concave, disease‐related, shape differences in the bilateral amygdala, hippocampus, accumbens, and thalamus. As there was a concordance of thinning and surface contraction, these surface changes may reflect losses of subvolumes relatively near the structural surfaces. In turn, we found a predominance of convex, disease‐related, shape differences in the bilateral putamen and the pallidum. Notably, the caudate showed a mix of both concave and convex shape differences. In the majority of these instances, differences in thinning/thickening and contraction/expansion were concordant (i.e., overlapping), which suggested to us that these changes more likely reflected variable subvolume changes relatively close to the structural surfaces.

The ranking of effect sizes (i.e., Cohen's *d*) corroborated the shape observations, and were consistent with prior volume‐based meta‐analyses (Haijma et al., 2013; van Erp et al., 2016). In a meta‐analysis of overlapping subjects, the volumes of the hippocampus, amygdala, accumbens, and the thalamus were reported to be smaller in individuals with schizophrenia compared to healthy control participants, while the volumes of the putamen and the pallidum were larger, and the volume of the caudate did not differ (van Erp et al., 2016). In a meta‐analysis of over 18,000 subjects, Haijma et al. also reported volume reductions in the hippocampus, amygdala, accumbens, and the thalamus for individuals with schizophrenia compared with healthy control participants, with no volumetric differences in the caudate and putamen, and enlargement in the pallidum (Haijma et al., 2013). Our meta‐analysis also revealed patterns of shape differences that were contiguous across neighboring structures (Figures S9–S11). For example, concave shape differences extended across the amygdala, hippocampus, accumbens, and thalamus, with larger differences crossing between the amygdala and hippocampus, between the hippocampus and thalamus, and between the caudate and accumbens (Figures S9 and S11). These shape differences confirmed findings previously reported by multiple individual shape studies; that is, in the anteriolateral (CA1) and subicular portions of the hippocampus (Csernansky et al., 2002; Lee et al., 2004; McClure et al., 2013), and anterior and pulvinar portions of the thalamus (Csernansky, Schindler, et al., 2004); see supplemental materials for a more in‐depth summary of literature findings.

Previous work has observed that when compared with healthy control participants, individuals with schizophrenia show smaller neuron sizes in the CA1 and subiculum subfields of the hippocampus (Arnold et al., 1995), reduced total number of neurons in the lateral nucleus of amygdala (Kreczmanski et al., 2007), and in the anteroventral, anteromedial, and mediodorsal nuclei and pulvinar of the thalamus (Dorph‐Petersen & Lewis, 2017; Young, Manaye, Liang, Hicks, & German, 2000). Microscopic reductions in neuron size or total number of neurons may manifest in macroscopical reductions in gray matter volume measured in MRI. Smaller neuronal size may reflect structural and/or metabolic compromise in the soma, or reduction in the synapses and dendrites that are metabolically supported (Arnold et al., 1995). Furthermore, post mortem studies of individuals with schizophrenia show reduced pre‐ and postsynaptic markers in hippocampal pyramidal neurons (Glantz & Lewis, 1997; Harrison, 1999b; Harrison & Eastwood, 1998), as well as shorter length, number, and complexity of dendritic processes (Garey et al., 1998; Rosoklija et al., 2000).

Regarding the caudate and putamen, *postmortem* studies have reported both larger (Beckmann & Lauer, 1997) as well as smaller disease‐related changes in total neuron number (Kreczmanski et al., 2007). Consistent with this mix of findings, we observed both convex and concave shape differences in these structures (Figure S11). In addition, the regions where convex or concave differences were found tended to be contiguous with the loss and expansion patterns observed in neighboring structures. For example, convex shape differences extended across the caudate and putamen, and adjoining regions, whereas the dorsal concave shape differences in the caudate extended across to the neighboring thalamus. Our observed shape differences are consistent with these patterns of neuronal deficits reported in *postmortem* studies of individuals with schizophrenia, suggesting that common or similar disease mechanisms may contribute to gray matter volume reduction across multiple subcortical regions.

With regard to shape asymmetry, we also observed patterns that extended across multiple, neighboring structures. One predominantly exaggerated asymmetry pattern was observed across the hippocampus, amygdala, and thalamus in individuals with schizophrenia compared to control participants, while a pattern of *diminished* asymmetry in individuals with schizophrenia encompassed the ventral striatum and ventral and dorsal thalamus. Exaggerated asymmetry patterns in schizophrenia across multiple structures have not been reported by previous, volume‐based meta‐analyses, perhaps due to their subtlety. In the aforementioned meta‐analysis of over 18,000 subjects, Haijma et al. reported no statistically significant differences in global left‐versus‐right subcortical volume effect sizes between individuals with schizophrenia and healthy control participants (Haijma et al., 2013), though left‐sided decreases in hippocampus and amygdala volumes were greater than the right‐sided decreases. In a meta‐analysis comparing 884 individuals with schizophrenia and 1,680 healthy control participants, Okada et al. also reported increased leftward asymmetry only for the pallidum volume in schizophrenia, but not for other subcortical structures (Okada et al., 2016). Our observed exaggerated asymmetry across the multiple subcortical structures, that is, the hippocampus, amygdala, and thalamus, may be partially attributable to a genetic mechanism that controls the development of cerebral asymmetry in schizophrenia (Brun et al., 2008; Crow et al., 1989; Satizabal et al., 2019; Thompson et al., 2001; van der Meulen et al., 2020). Diminished asymmetry such as the effects we observed in the ventral striatum and ventral and dorsal thalamus might be a result of epigenetic dysregulation (Abdolmaleky et al., 2019). Environmental insults during neurodevelopment could also lead to disturbed brain asymmetry observed later in life (Algan & Rakic, 1997; Schindler et al., 2002). In addition, abnormal developments in neuroanatomical asymmetry may underlie the disturbed asymmetry patterns in brain network connectivity in individuals with schizophrenia (Gomez‐Gastiasoro et al., 2019; Guo, Han, Li, & Reddick, 2019; McKenna, Babb, Miles, Goff, & Lazar, 2020; Wang et al., 2019). Ongoing studies of populations of subjects at genetic risk for developing schizophrenia, such as relatives of patients with schizophrenia (de Zwarte et al., 2019; Harms et al., 2007; Mamah et al., 2008; Zhu et al., 2018), may help elucidate the specific roles of genetic influence and environmental factors that contribute to the development of schizophrenia.

The brain's deeper structures are integral components of distributed functional network circuitry. In particular, the thalamus and striatum have direct, excitatory connections with the cortex, while the pallidum receives inhibitory signals from the striatum and limbic structures, and in turn, passes inhibitory signals to the thalamus. Information from different cortical areas, including the prefrontal, frontal, motor, and sensory areas, is received by the striatum via excitatory projections, and then passed on to the thalamus via inhibitory projections from the pallidum. The thalamus projects to the cortex, completing a cortico‐basal ganglia‐thalamo‐cortical loop (Kandel, Schwartz, & Jessell, 1991). The hippocampus and amygdala also have reciprocal excitatory connections with the medial prefrontal cortex (Barbas & Blatt, 1995; Carmichael & Price, 1995), and project to the anterior nucleus of the thalamus as part of Papez' circuit (Jankowski et al., 2013). Structural neuroimaging studies have demonstrated that schizophrenia is associated with widespread gray matter reduction in the brain, predominantly in medial temporal, frontal, and parietal cortical regions, as well in the midline limbic structures and the deep thalamic and striatal nuclei (Bora et al., 2011; Fornito, Yucel, Patti, Wood, & Pantelis, 2009; Honea, Crow, Passingham, & Mackay, 2005; Keshavan, Prasad, & Pearlson, 2007; Lawrie & Abukmeil, 1998; Levitt et al., 2010; McCarley et al., 1999; Palaniyappan, Balain, & Liddle, 2012; Pantelis et al., 2009; Pearlson & Calhoun, 2007; Pearlson & Marsh, 1999; Schmitt, Hasan, Gruber, & Falkai, 2011; Shenton, Dickey, Frumin, & McCarley, 2001; Shenton, Whitford, & Kubicki, 2010). These multifocal brain abnormalities are hypothesized to reflect a “fragmentation of brain pathologies” that ultimately leads to breakdowns in network organization (Shenton et al., 2001; van den Berg, Gong, Breakspear, & van Leeuwen, 2012). Advances in structural and functional neuroimaging studies continue to provide evidence that schizophrenia is a disorder with subtle, multifocal abnormalities involving distributed changes in brain network architecture (Wang & Csernansky, 2014). Our shape meta‐analysis findings help us understand this network disorganization in schizophrenia by isolating the specific locations of structural subvolume abnormalities on the surface of deep‐brain structures to identify which afferent and efferent connections are affected (Bullmore, Frangou, & Murray, 1997; Friston & Frith, 1995; Meyer‐Lindenberg et al., 2001; Volkow et al., 1988; Weinberger, Berman, Suddath, & Torrey, 1992).

Focal subcortical shape deformities in individuals with schizophrenia, such as the anterior‐lateral, posterior‐lateral aspects of the hippocampus, are known to correlate with focal thinning of cortical regions involved in both dorsal and ventral visual processes (Qiu et al., 2010), as well as lower white matter integrity as measured by fractional anisotropy (Qiu et al., 2010). These findings are consistent with cellular studies showing that the number of neurons expressing NADPH‐d enzyme is lower in the hippocampus (Akbarian et al., 1993) as well as in the dorsolateral prefrontal cortex (Akbarian et al., 1993), of subjects with schizophrenia, suggesting the presence of a common pathological process that has interrupted normal neurodevelopment (Akbarian, Vinuela, et al., 1993). Cobia et al. (2017) showed that inward deformity of thalamic shape in the pulvinar region correlated with reduced thickness of the frontal, temporal and parietal cortices, and longitudinal changes in these thalamic and cortical subregions were similarly correlated. The thalamic surface zones reported by Cobia et al. (2017) strongly overlapped with thalamic regions that were the thinnest in our meta‐analysis. The pulvinar of the thalamus has broad distributions of connections across the association cortex (Sherman & Guillery, 2011), and its abnormalities were found to be related to cortical abnormalities in individuals with schizophrenia (Sherman & Guillery, 2011). Therefore, our shape findings provide further structural evidence that the development of multiple brain structures are affected in schizophrenia, which support changes in functional brain networks.

Our shape meta‐analysis found that higher chlorpromazine dose equivalents were related to concave shape differences primarily in the hippocampus and amygdala, but also in the caudate, accumbens, and thalamus. The literature on the association between subcortical structure and second‐generation antipsychotic treatment reflects conflicting findings. In van Erp et al., where an overlapping sample was analyzed for subcortical volumes (van Erp et al., 2016), no relationship was found between chlorpromazine dose equivalents and differences in subcortical volumes. However, the volumetric meta‐analysis by Haijma et al. (2013) found that a higher dose of atypical antipsychotics was associated with larger caudate volumes. While some studies have found that antipsychotics were related to increases in gray matter volume or attenuated volume reduction in subcortical structures (see Konradi & Heckers, 2001; Roiz‐Santianez et al., 2015 for reviews), others have reported that volume reductions in the hippocampus (Li et al., 2018) and caudate (Roiz‐Santianez et al., 2014) were associated with antipsychotic drug treatment (Dorph‐Petersen et al., 2005; Ho et al., 2011; Roiz‐Santianez et al., 2015). In a shape analysis by Mamah et al. (2012), schizophrenia patients who were taking olanzapine showed inward shape changes in the hippocampus as compared with healthy controls, but changes that were relatively smaller than inward shape observed in patients taking first‐generation antipsychotics. In a meta‐analysis of 778 schizophrenia patients, a longer duration of illness was correlated with increased globus pallidus volumes (Hashimoto et al., 2018). In rodent studies, antipsychotic drugs have been shown to affect synaptic ultrastructure, but not neuronal number or size. Atypical antipsychotics tended to upregulate dendritic spine formation and synaptogenesis, whereas typical antipsychotic drugs tended to downregulate them or have no significant effect (Critchlow, Maycox, Skepper, & Krylova, 2006; Delotterie et al., 2010). Following atypical antipsychotic treatment, increased synapses have been observed in the striatum (see Harrison, 1999a for a review) and the hippocampus (Park et al., 2013), whereas typical antipsychotics were not associated with similar changes (Critchlow et al., 2006). Further examination of the mechanisms of antipsychotic drugs' effects on brain structures may help explain the findings of relationships between drug classes and brain changes (i.e., concave shape differences or volume reductions associated with higher doses of first‐ or second‐ generation drugs) (Li et al., 2018).

Our shape meta‐analysis showed that higher positive symptom severity (SAPS total) scores were associated with concave shape differences in the hippocampus, amygdala, caudate, and thalamus. In the abovementioned volumetric meta‐analysis in an overlapping sample (van Erp et al., 2016), no relationships between subcortical volumes and severity of positive or negative symptoms were found. Moreover, in the literature, the association between subcortical structure and psychopathological symptoms has been largely absent (Gong, Lui, & Sweeney, 2016; Haukvik, Hartberg, & Agartz, 2013; Levitt et al., 2010; Li et al., 2018). While the inconsistent relationship in the literature suggests that subcortical structures may not reflect the intensity of psychopathology (Gong et al., 2016), our findings are consistent with the few studies that have shown a relationship between increased positive symptom severity and reduced accumbens volume (Mamah et al., 2007), longitudinal thalamic shape deformation (Wang et al., 2008), and reductions in cortical gray matter measures (Chen et al., 2014).

Future studies in structural shape should consider the following limitations with respect to our particular analytic tools: In the hippocampus, we found concave changes more prominently in the lateral (proximal to the CA1 hippocampal subfield) and medial regions (proximal to the subiculum). These patterns were more posterior than the more anterior shape deformities reported by groups using diffeomorphic surface mapping methods (Csernansky et al., 2002; Qiu et al., 2010). One explanation may be that the FreeSurfer software, used by every site in our meta‐analysis, generates subcortical gray matter boundaries that are sometimes noisy (Wang et al., 2009). While this noise may not affect whole‐structure volume measurements (see volume meta‐analysis paper by van Erp et al. (2016), it may influence shape measurements such as ours, based on vertex‐wise information. The nature of the meta‐analysis in this study did not permit the implementation of methodologies that could have generated smooth subcortical surfaces. In addition, harmonization of heterogeneous clinical variables (such as medication, psychopathological symptoms) is challenging, given the differences in outcome measures/variables used.

There is a growing literature of subtypes of schizophrenia. Using MRI, EEG, ocular motor, and cognition measures, the B‐SNIP consortium reported three biotypes that differed on social‐relational negative symptoms (Clementz et al., 2020). We and others have reported subtypes as defined by the degree of neuropsychological impairment who differed also in their cortical neuroanatomy (Cobia, Csernansky, & Wang, 2011). McCutcheon, Abi‐Dargham, and Howes (2019), however, reported no evidence of subtypes based on treatment responses in a meta‐analysis. The ENIGMA consortium provides an ideal setting for future, prospective meta‐analytical studies to define and validate subtypes of schizophrenia based on genetics, cognition and other biomarker measures (Smeland et al., 2017; T. Wang et al., 2017). Using polygenic risk scores (PGRS), a recent review of associations between functional MRI activity and polygenic risk for schizophrenia reports that although genetic modulation on brain function for schizophrenia occurs predominantly in frontal areas, it also impacts the task‐dependent recruitment across multiple brain regions (Purcell et al., 2009). The ENIGMA consortium is therefore also ideal for meta‐analytic studies that can provide anatomical and connectomic mechanisms for the widespread cognitive deficits observed in schizophrenia (Smeland et al., 2017). Further, while antipsychotic drugs and behavioral treatments have not been effective in relieving negative symptoms and cognitive impairment in schizophrenia (Green, Kern, & Heaton, 2004; Nuechterlein et al., 2011), cognitive behavior therapy could lead to increased activation in the thalamus (Kumari et al., 2010) (see Howells, Baldwin, and Kingdon (2016) for a review). Treatment modalities such as aerobic exercise have been shown to improve positive and negative symptoms and quality of life (Dauwan, Begemann, Heringa, & Sommer, 2016; Firth, Cotter, Elliott, French, & Yung, 2015; Vancampfort, Rosenbaum, Ward, & Stubbs, 2015). Aerobic exercise has been demonstrated to improve a range of cognitive functions such as working memory and verbal learning (Vakhrusheva, Marino, Stroup, & Kimhy, 2016) and brain structure (Kandola, Hendrikse, Lucassen, & Yucel, 2016; Malchow et al., 2016; Pajonk et al., 2010) among individuals with schizophrenia. The mechanism by which aerobic exercise renders these beneficial effects are not established but is reportedly related to improvement in neurogenesis and neuroplasticity processes, particularly in the hippocampus (Falkai et al., 2013; Vakhrusheva et al., 2016). Therefore, future studies may provide further mechanistic basis and targets for potential interventions.

Finally, as data were merged from multiple sites, we chose a statistical design based on meta‐analysis to generate consensus maps from the various data sets analyzed. Other approaches are possible, and in Radua et al. (2020) we compared meta‐analysis with mega‐analysis, in which individual level data from all sites are centralized, and site‐level effects are be modeled as random effects. In a meta‐analysis, which we used here, it is not assumed that the data from each site have the same mean and variance, as models are fitted locally and effect sizes are combined, with a site‐dependent weight. Although we did not do this here, additional adjustments can be made to the data from each site to further harmonize it, including ComBat and its variants (Fortin et al., 2018; Pomponio et al., 2020), hierarchical Bayes models (Kia et al., 2020), and generative adversarial networks (Liu et al., 2021). It is still an open question how to adjust site‐level data to increase the distributional overlap across sites, especially when inclusion criteria differ somewhat and latent confounds at each site can lead to under‐ or overadjustment of data (Kia et al., 2020). As such we used a meta‐analysis, which gives comparable results to other methods in recent comparisons (Radua et al., 2020).

In conclusion, our meta‐analysis of subcortical shapes revealed strong patterns of shape differences and shape asymmetry differences that provide finer‐scale information than prior studies of structural volumes, and spanned multiple neighboring subcortical structures. Findings from our shape meta‐analysis suggest that common mechanisms may contribute to gray matter reduction across multiple subcortical regions, which may enhance our understanding of the nature of network disorganization in schizophrenia.

## 5 COLLABORATORS

The authors also would like to acknowledge the following members of the Karolinska Schizophrenia Project (KaSP) Consortium: Lars Farde, Centre for Psychiatry Research, Department of Clinical Neuroscience, Karolinska Institutet, & Stockholm Health Care Services, Stockholm County Council, Stockholm, Sweden; Lena Flyckt, Centre for Psychiatry Research, Department of Clinical Neuroscience, Karolinska Institutet, & Stockholm Health Care Services, Stockholm County Council, Stockholm, Sweden; Helena Fatouros‐Bergman, Centre for Psychiatry Research, Department of Clinical Neuroscience, Karolinska Institutet, & Stockholm Health Care Services, Stockholm County Council, Stockholm, Sweden; Simon Cervenka, Centre for Psychiatry Research, Department of Clinical Neuroscience, Karolinska Institutet, & Stockholm Health Care Services, Stockholm County Council, Stockholm, Sweden; Karin Collste, Centre for Psychiatry Research, Department of Clinical Neuroscience, Karolinska Institutet, & Stockholm Health Care Services, Stockholm County Council, Stockholm, Sweden; Pauliina Victorsson, Centre for Psychiatry Research, Department of Clinical Neuroscience, Karolinska Institutet, & Stockholm Health Care Services, Stockholm County Council, Stockholm, Sweden; Göran Engberg, Department of Physiology and Pharmacology, Karolinska Institutet, Stockholm, Sweden; Sophie Erhardt, Department of Physiology and Pharmacology, Karolinska Institutet, Stockholm, Sweden; Lilly Schwieler, Department of Physiology and Pharmacology, Karolinska Institutet, Stockholm, Sweden; Anna Malmqvist, Department of Physiology and Pharmacology, Karolinska Institutet, Stockholm, Sweden; Mikael Hedberg, Department of Physiology and Pharmacology, Karolinska Institutet, Stockholm, Sweden; Funda Orhan, Department of Physiology and Pharmacology, Karolinska Institutet, Stockholm, Sweden; Carl M Sellgren, Department of Physiology and Pharmacology, Karolinska Institutet, Stockholm, Sweden; Fredrik Piehl, Neuroimmunology Unit, Department of Clinical Neuroscience, Karolinska Institutet, Stockholm, Sweden; Ingrid Agartz, NORMENT, Division of Mental Health and Addiction, Oslo University Hospital & Institute of Clinical Medicine, University of Oslo.

## CONFLICT OF INTERESTS

One of the authors (TGMvE) has had a research contract with Otsuka Pharmaceutical. One of the authors (AP) has served as a consultant for Boehringer Ingelheim. One of the authors (DJS) has received research grants and/or honoraria from Lundbeck and Sun. One of the authors (DHM) has served as a consultant for Boehringer Ingelheim, Aptinyx, and Greenwich Biosciences. One of the authors (SC) has received grant support from AstraZeneca as co‐investigator, and has served as a speaker for Otsuka Pharmaceuticals. Authors PMT, CRKC, and NJ received a research grant from Biogen, Inc. (Boston) for research unrelated to this manuscript. The remaining authors declare no potential conflict of interest.

## Supporting information


**Appendix S1**: Supporting Information

## Data Availability

Data sharing is not applicable to this article as no new data were created or analyzed in this study.
