# Supplementary material for: A meta‐analysis of deep brain structural shape and asymmetry abnormalities in 2,833 individuals with schizophrenia compared with 3,929 healthy volunteers via the ENIGMA Consortium
Source: Hum Brain Mapp. 2021 Sep 8;43(1):352–72. doi: 10.1002/hbm.25625 (PMC8675416; doi:10.1002/hbm.25625)
Supplement: Supplementary file 1 — Appendix S1: Supporting Information [file HBM-43-352-s001.docx]

**Supplemental Materials for**

**A Meta-Analysis of Deep Brain Structural Shape and Asymmetry Abnormalities in 2,833 Individuals with Schizophrenia Compared to 3,929 Healthy Volunteers via the ENIGMA Consortium**

**Additional Materials & Methods**

Data

**Figure S1** shows the 24 worldwide cross-sectional study samples drawn from 21 institutions (sites). Also included are the University of Southern California and Institute of Illinois Technology as data analysis site. The QTIM atlas data used in this study was collected in Brisbane, Australia (not shown on map). **Table S1** shows sample sizes, demographic, and clinical variables for all participating study samples. **Table S1** also shows the FreeSurfer version used to analyze the data across the samples, with the majority using v5.3.0 (17/24), followed by v.6.0 (3), v5.1.0 (2), v4.5.0 (1) and v4.0.1 (1).

Statistical meta-analysis

The meta-analyses pooled each sample’s Cohen’s *d* group effect sizes, for each vertex and shape measure (log Jacobian and thickness), using an inverse variance-weighted random-effects model as implemented in the R package *metafor* (version 1.9-1) (Viechtbauer, 2010). Random effects models, compared to fixed effect models, do not make the assumption of a true (the same) effect size for each study. True within-study mean effect is itself treated as a random variable drawn from a distribution. In this way, random effects meta-analysis controls for study-specific differences (e.g., mean age) by separating overall variance into between- and within-study components. By weighing the variance components in the pooled effect size estimates, this approach protects against dominating effects of the largest samples in the meta-analysis (Borenstein, Hedges, Higgins, & Rothstein, 2011) (p. 77). The random-effects models were fit using the restricted maximum likelihood method (Harville, 1977); for additional model details see (Cooper, Hedges, & Valentine, 2009).

In addition to Cohen’s *d* effect size estimates, *metafor* computes other measures, including standard errors, p-values, confidence intervals (CIs), and measures of heterogeneity (e.g., I^2^). I^2^ (100% ´ (Cochran’s Q - df) / Cochran’s Q) describes the percent variance across studies that is associated with heterogeneity rather than chance and has the principal advantage over other methods of heterogeneity estimation in that it is independent of a) the size of the meta-analysis, b) the types of studies included in the meta-analysis, and c) the outcome data used in the meta-analysis and hence can readily be compared across meta-analyses studies (Higgins, Thompson, Deeks, & Altman, 2003). I^2^ values of 0%, 25%, 50%, and 75% are considered reflective of no, low, moderate, and high variability/heterogeneity in effect size estimates across studies (Higgins et al., 2003).

**Additional Results**

Main effects of diagnosis

In the following, we describe the spatial patterns of these effects in each individual structure.

1. **Hippocampus** (**Figure S2**): We found thinning in the lateral and anterior regions, most pronounced in the lateral, medial portions of the head, body and tail of the hippocampus. This pattern suggests the involvement of the CA1 and subicular subregions. Similar patterns of shape abnormalities have been observed in prior studies (Csernansky 1998, 2002). Surface contraction is observed along almost the entire structure, most pronounced along the lateral aspects, suggesting involvement of the CA1 subfield.
2. **Amygdala** (**Figure S3**): Thinning is observed along the lateral surface of the amygdala, whereas pronounced surface contractions also include medial portions of the surface in addition to the lateral side.
3. **Putamen** (**Figure S4**): Increased thickness is observed along the superior, inferior and some anterior aspects, while a smaller amount of thinning can be seen in the central regions of the medial and lateral aspects. Surface expansion follows similar patterns, with fewer areas of contraction.
4. **Accumbens** (**Figure S5**): Thinning is observed on the anterior and especially inferior aspects, and surface contraction along almost the entire structure.
5. **Pallidum** (**Figure S6**): Both greater thickness and surface expansion can be seen along almost the entire structure, most pronounced along the superior and inferior aspects.
6. **Caudate** (**Figure S7**): Greater thickness is observed along the superior, inferior and some anterior aspects, while thinning can be seen in the central medial aspect along the length of the structure. Surface expansion follows similar patterns while no surface contraction is observed.
7. **Thalamus** (**Figure S8**): Thinning is observed throughout the structure, most pronounced in the dorsoanterior and posterior regions, suggesting involvement in the anterior and pulvinar subnuclei. Similar patterns of shape abnormalities have been reported before (Csernansky 2002, Cobia 2017). However, contrary to previous findings, the mediodorsal regions of the thalamus do not show inward deformity (thinning). Surface contraction is observed along most of the structure.
8. **Hippocampus, amygdala, thalamus** (**Figure S9**): The left hemisphere is shown as a representative for clarity without views being obstructed from the right hemisphere. Patterns are similar across both hemispheres. Thinning and contraction occur across the hippocampus, amygdala, and thalamus; thinning and contraction patterns are similar.
9. **Basal ganglia** (caudate, accumbens, putamen, pallidum, **Figure S10**): Left hemisphere is shown, as noted above. Thickening and surface expansion are occurring across the lenticular nucleus (caudate, putamen and pallidum) in a contiguous fashion. The thinning pattern extends across both the caudate and accumbens. These patterns suggest anatomically extended effects that affect several neighboring structures together. See also **Figure S10**, which shows all left-hemisphere subcortical structures together.
10. **All subcortical structures** (hippocampus, amygdala, thalamus, caudate, accumbens, putamen, pallidum, **Figure S11**): Left hemisphere is shown, as noted above. We observe a thinning pattern that spans the amygdala, hippocampus, thalamus, caudate and accumbens (left column). The patterns of surface contraction are similar (right column). Increased thickness and surface area expansion can be seen across the globus pallidum and putamen (left and right columns, respectively).

Shape asymmetry

Here we make further interpretations on whether the observed asymmetry differences indicated a leftward vs. rightward, or an exaggerated vs. diminished normal symmetry. This was done in combination with examinations of group differences across both hemispheres.

For the hippocampus, amygdala, and the accumbens, the thickness and regional surface area were reduced across both hemispheres (**Figures S1,2,4**) for patients, compared to controls. Therefore, an increased asymmetry index would suggest a leftward exaggeration of the normal asymmetry for the SCZ group in these structures, compared to controls.

For the putamen, the thickness and regional surface area were predominantly increased across both hemispheres; small areas showed decreases (**Figures S3**) for SCZ, compared to controls. Further, areas showing differences in symmetry indices overlap with areas showing increased group differences but not decreases. Therefore, an increased asymmetry index would suggest a rightward exaggeration of the normal asymmetry, in the putamen, for SCZ compared to controls.

For the thalamus, the case is different for the thickness vs. surface area asymmetry. The thickness asymmetry index was predominantly smaller for SCZ, compared to controls, whereas the surface area asymmetry index showed similar proportions of increases and decreases for SCZ. For thickness, the thalamus showed bilateral decreases for SCZ, compared to controls (**Figure S7**). Therefore, a lowered asymmetry index would suggest a diminished normal asymmetry for SCZ in the thalamus, compared to controls. On the other hand for the regional surface area, with the thalamus showing bilateral decreases for SCZ as compared to CON (**Figure S7**), a mostly increased asymmetry index would suggest leftward exaggeration of the normal asymmetry for SCZ in the thalamus as compared to controls.

Quality control

The shape model quality of each region of interest for each subject was classified as (1) unacceptable, (2) acceptable, or (3) ideal. Human raters were blind to all participant information. The correction was simply to exclude observations with unacceptable model quality, which indicated quality control (QC) failure, from the statistical analysis. This was done separately for each ROI, i.e. each regional map is based on a slightly different set of subjects. Quality rating and exclusion was performed at the primary-analysis level at each site; QC information was not directly incorporated at the meta-analysis stage. Rates of QC failure ranged between 0.5% and 3.4% depending on the diagnostic group and region (**Table S2**). The proportions of failure were not significantly different between SCZ and HC groups based on the Log-Odds Ratio test in all regions except the left accumbens after Bonferroni correction.

Shape measure interpretation

We assume in general a cigar-shaped object as a stereotypical shape in our analyses. A cigar may be compressed by being shortened along its main axis without changing its width. This is an example of contraction without thinning. On the other hand, we can stretch a cigar along its main axis in a manner that preserves its surface area. The cigar would get thinner and longer. This would be an example of thinning without (isotropic) surface contraction (**Figure S12 Panel C**).

It is worth noting that these exact scenarios are unlikely. Medial thickness and surface area are different, but they are generally coupled. A more realistic scenario is that local shape differences stem from local volume differences. In this more common case, a “thinning” will correspond to a contraction where the surface is convex, and to an expansion where the surface is concave. The converse correspondence can be observed as well: thickening implies expansion if the surface is convex and contraction otherwise. This is seen in **Figure S12 Panel B** and in our main results in the putamen and caudate.

**Supplemental Figures**

**Figure S12. Shape Measure Interpretation. (A)** A volume-preserving shape change in a hippocampal mesh model, shrinking the tail and expanding the head of the hippocampus. **(B)** The thickness difference and logarithm of the surface Jacobian corresponding to (A). The signs of the two measures are the same over convex regions (majority of the surface), but opposite in concave regions (part of the head and tail). **(C)** Stereotypical shape differences with respect to an atlas model resulting in compression but no thickness change (left) and thinning but no Jacobian change (right).


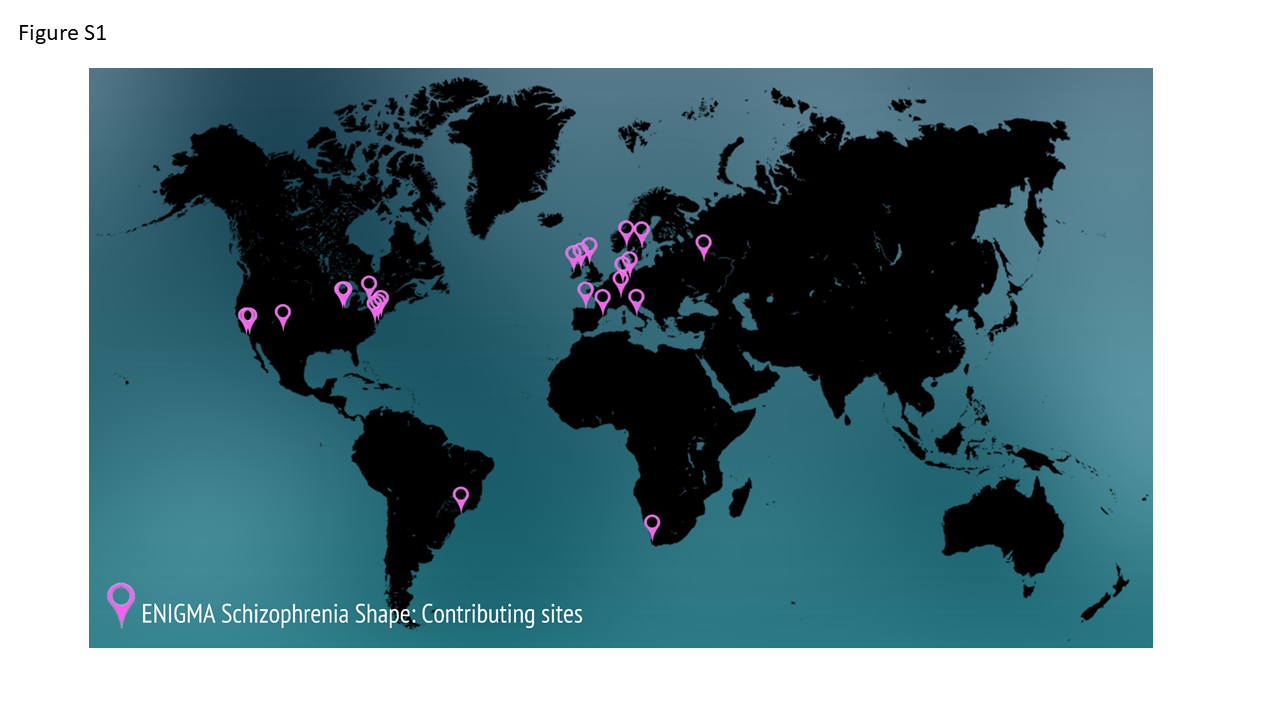

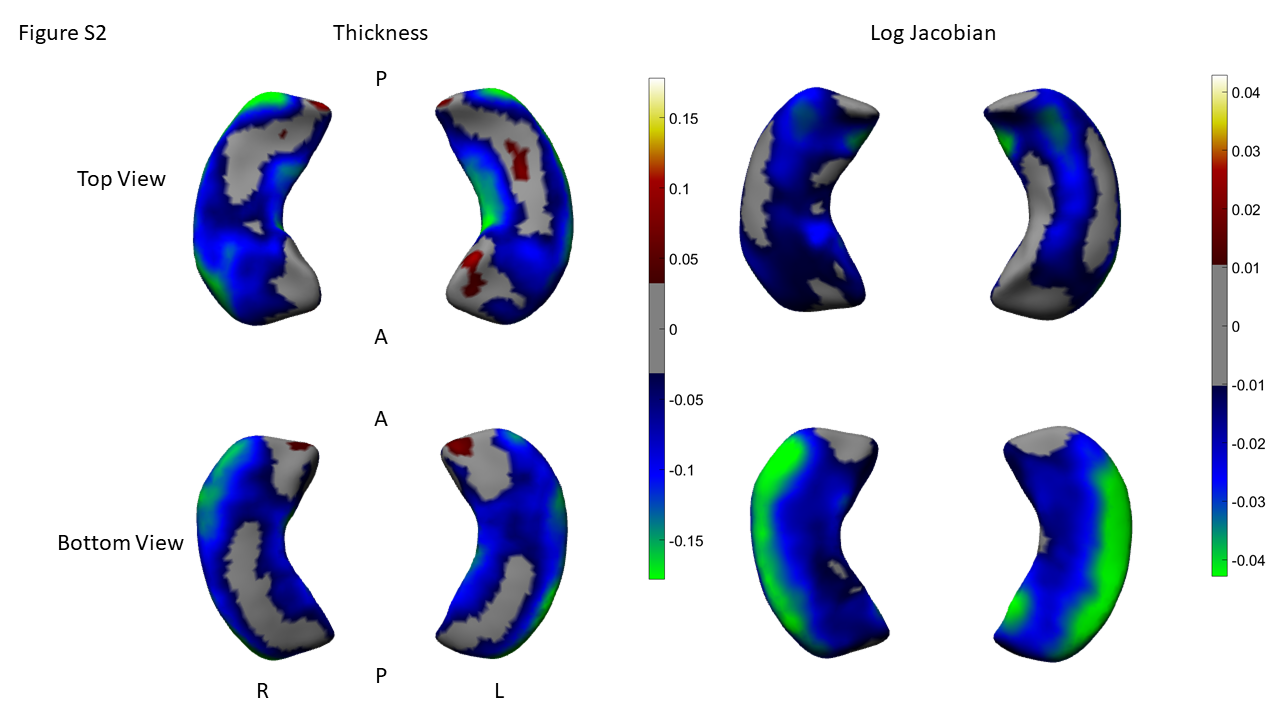

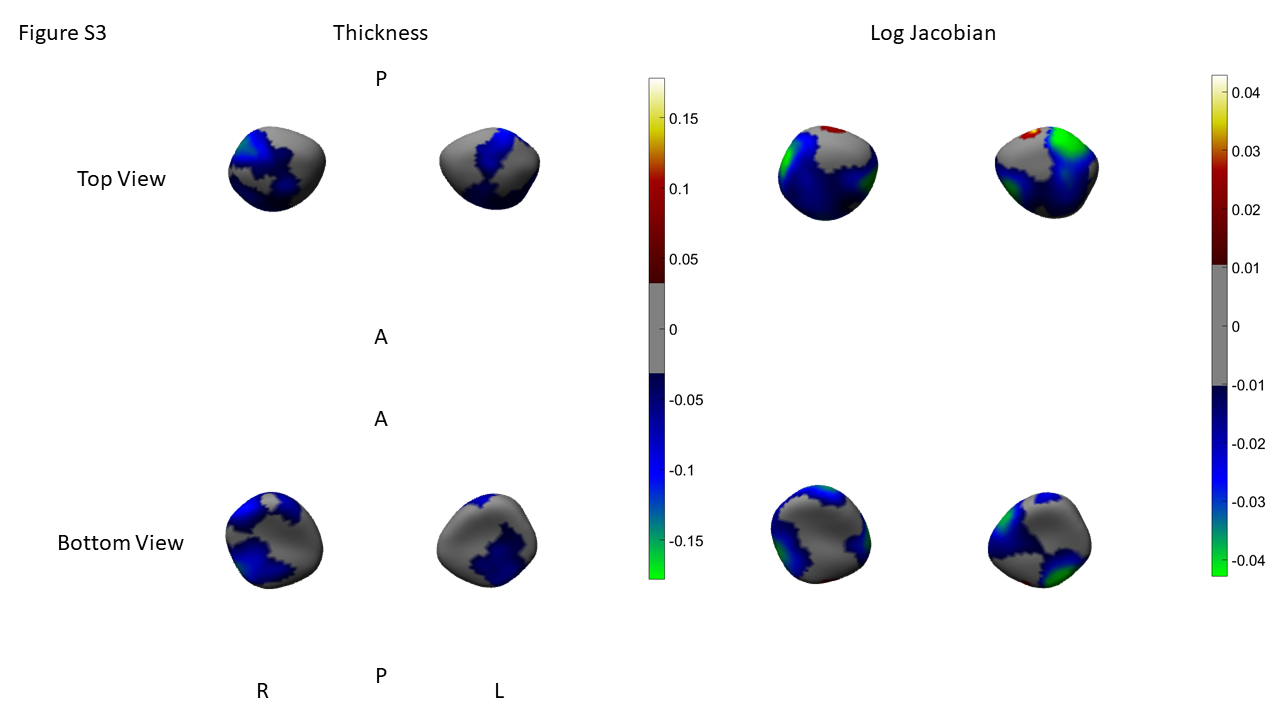

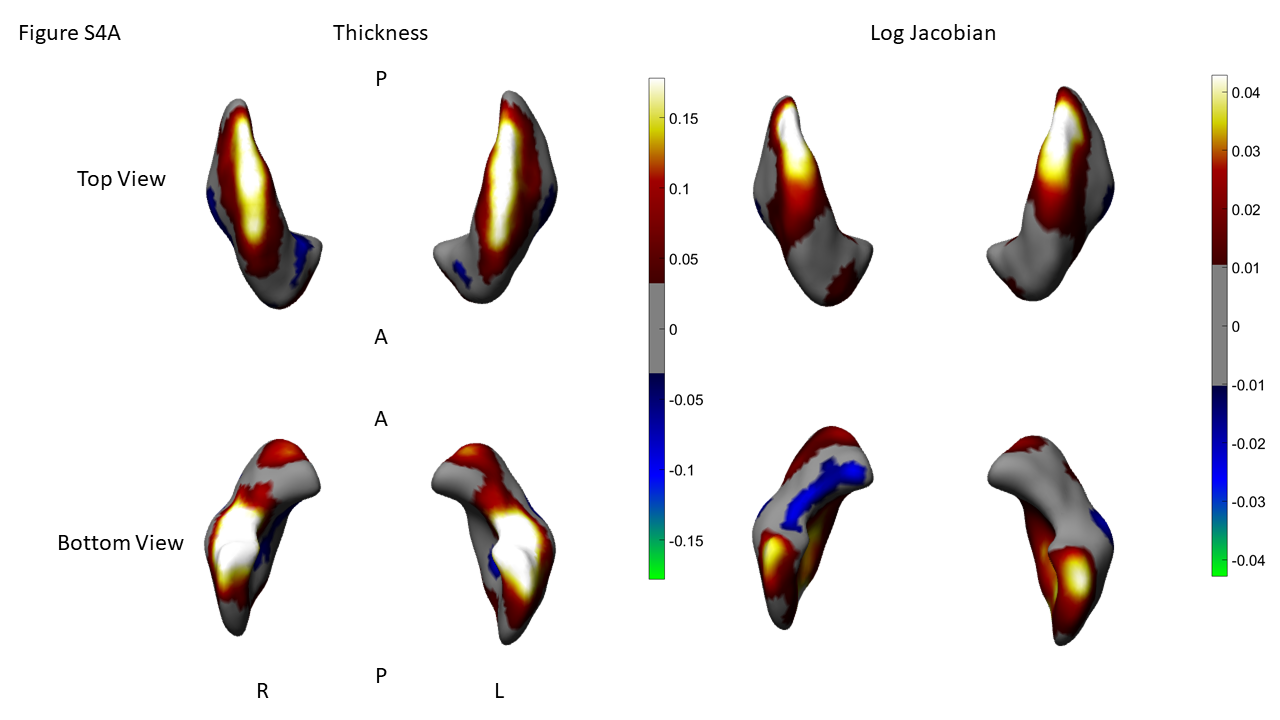

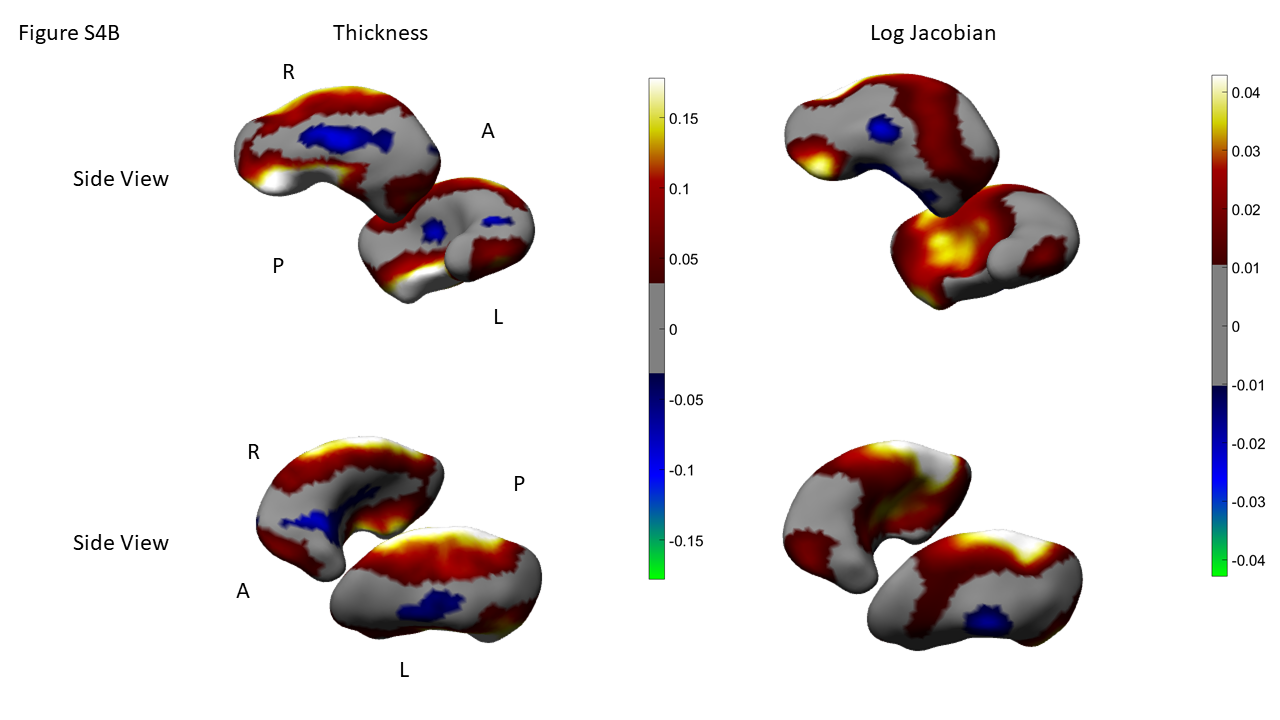

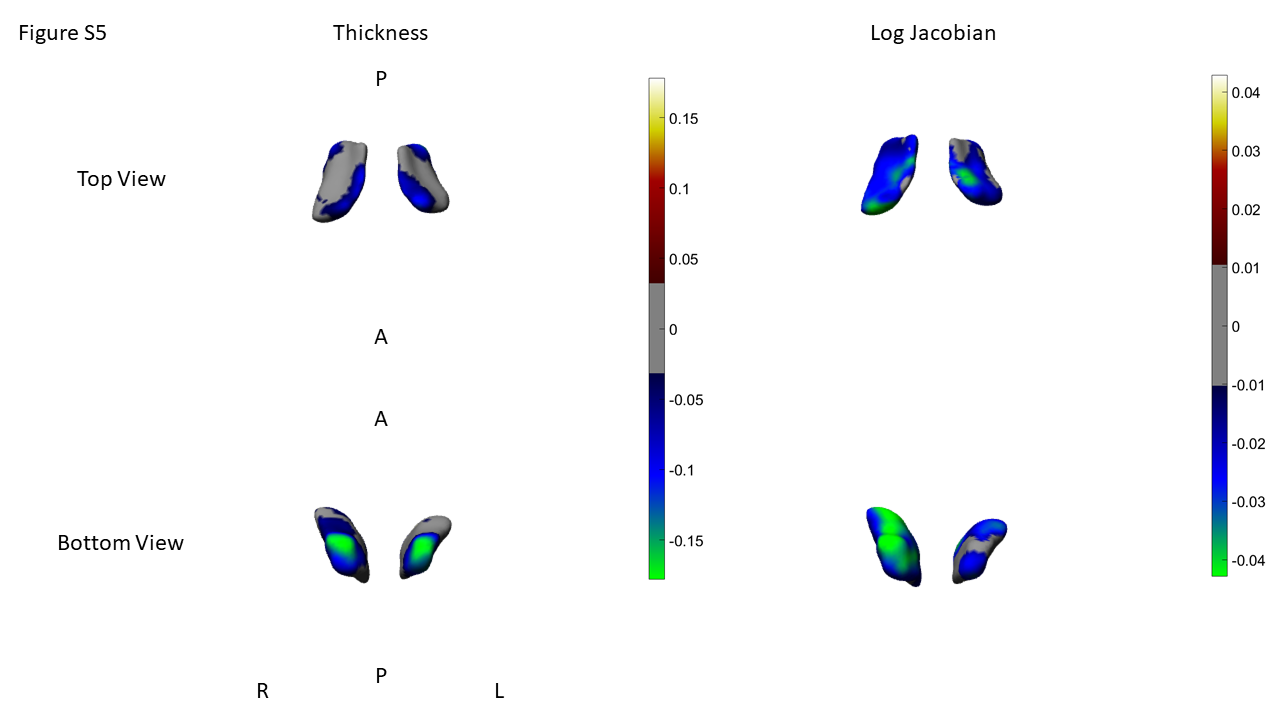

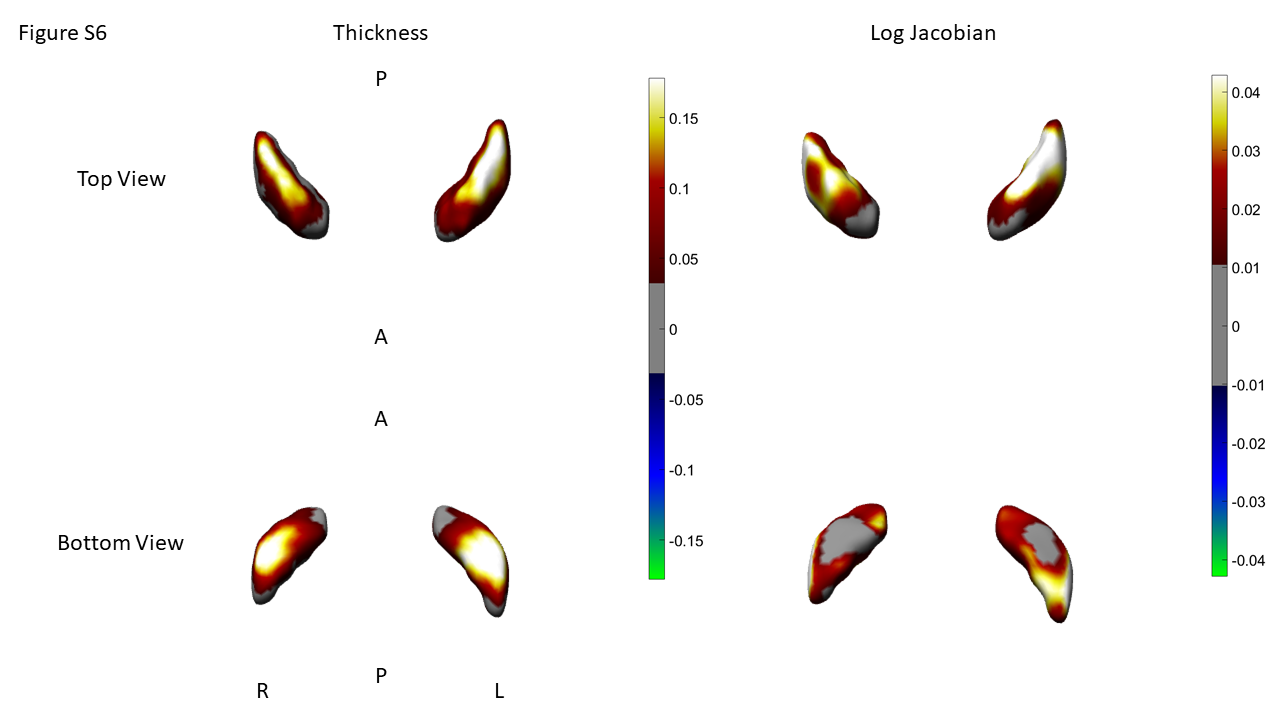

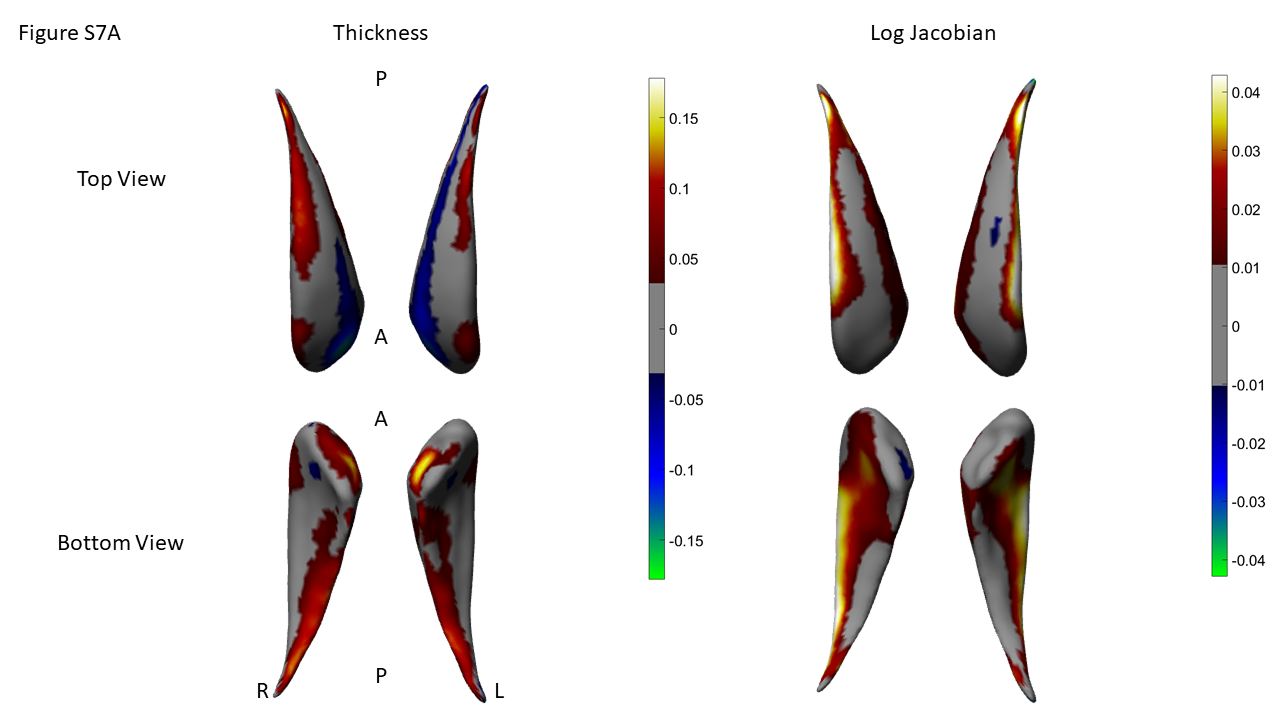

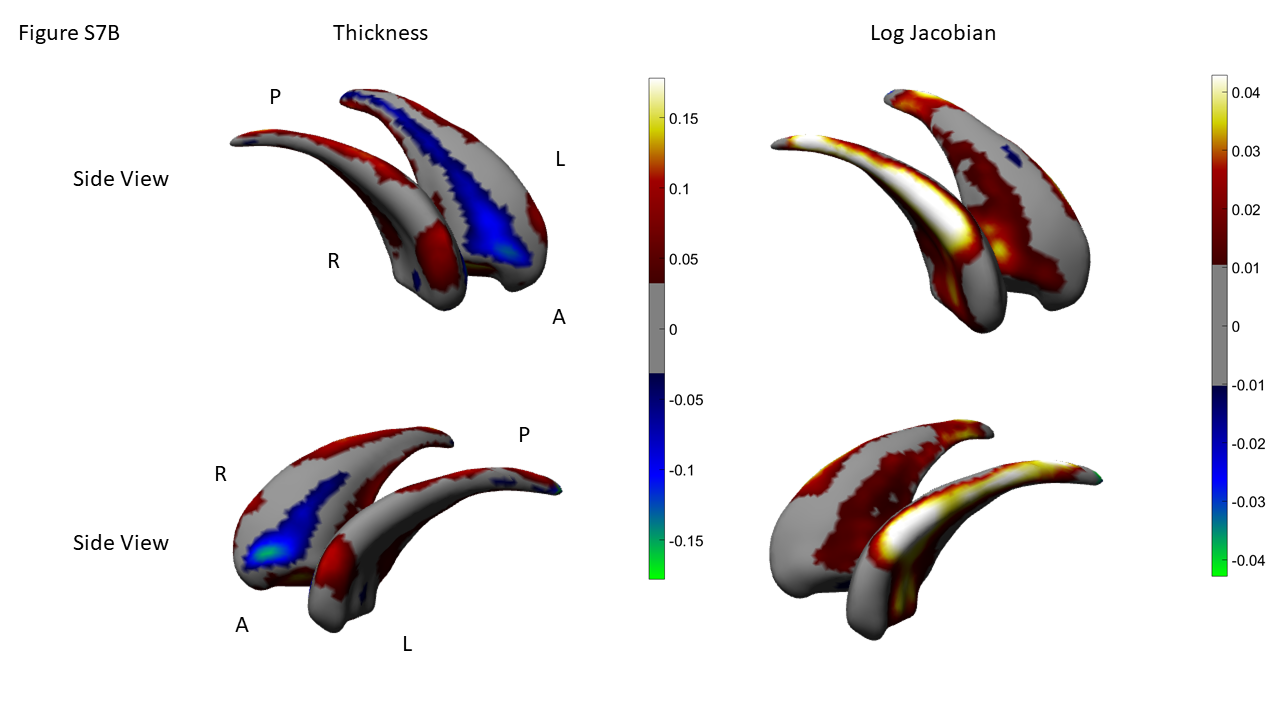

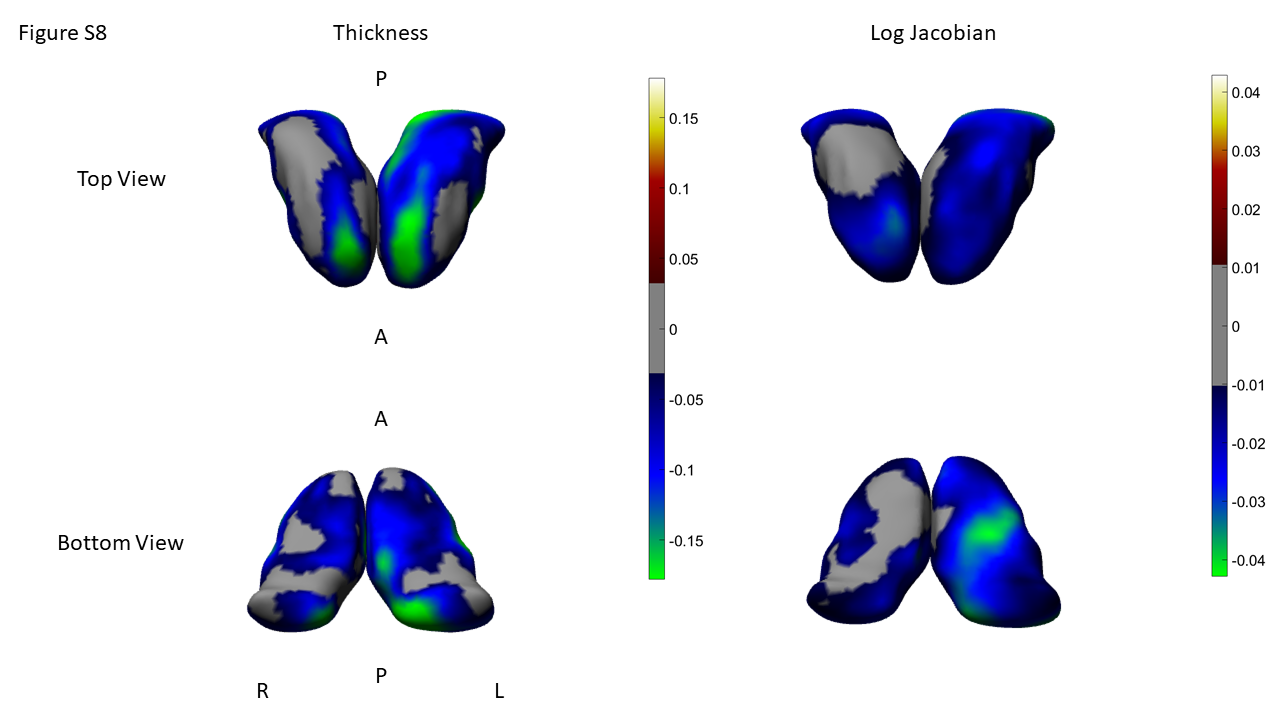

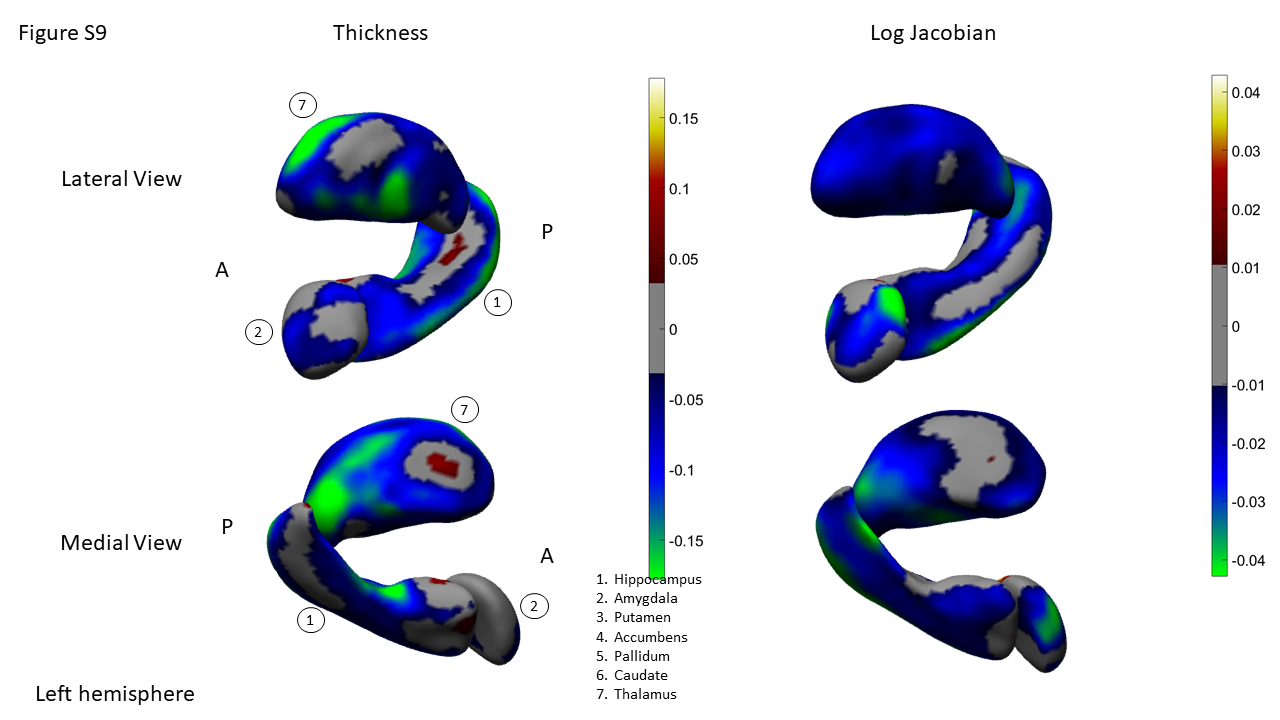

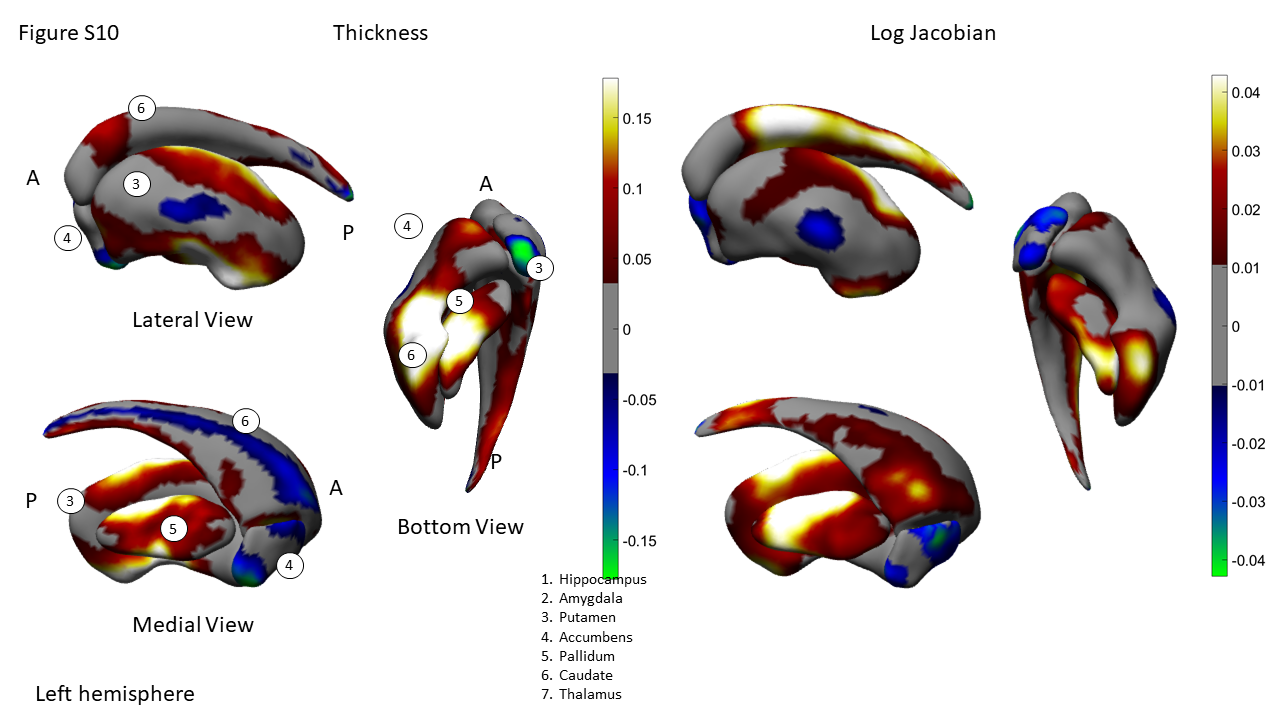

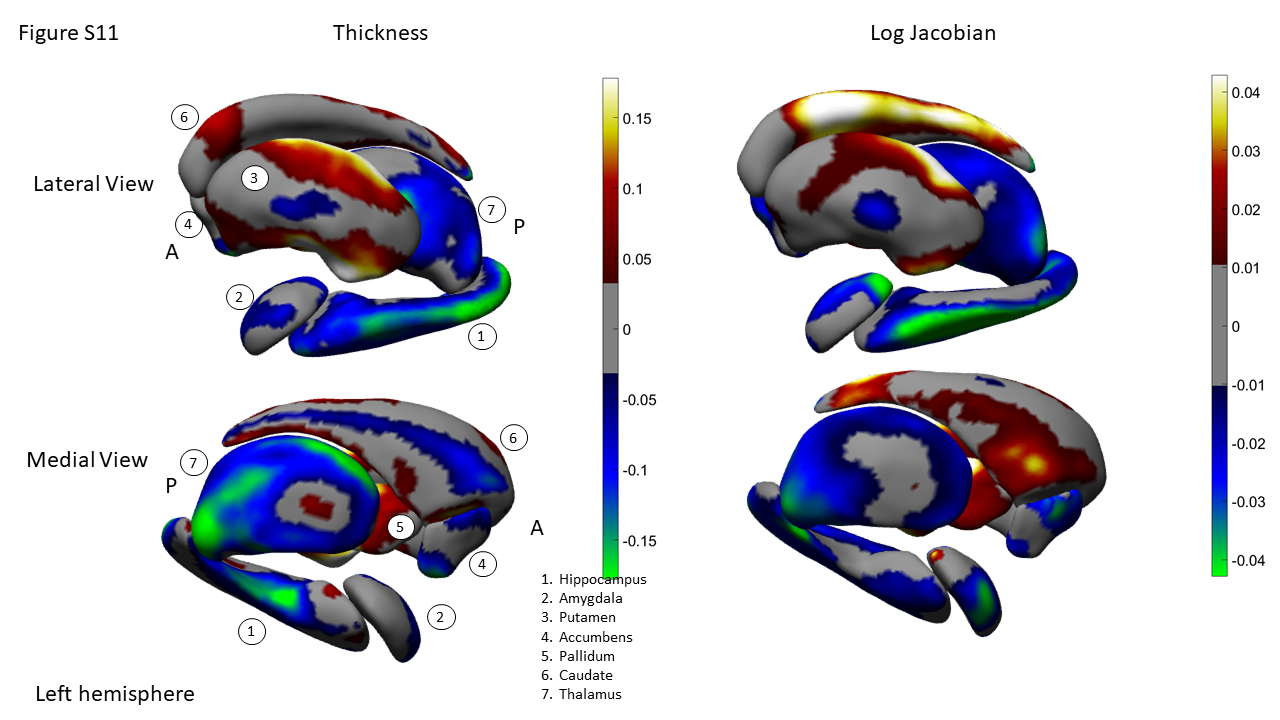

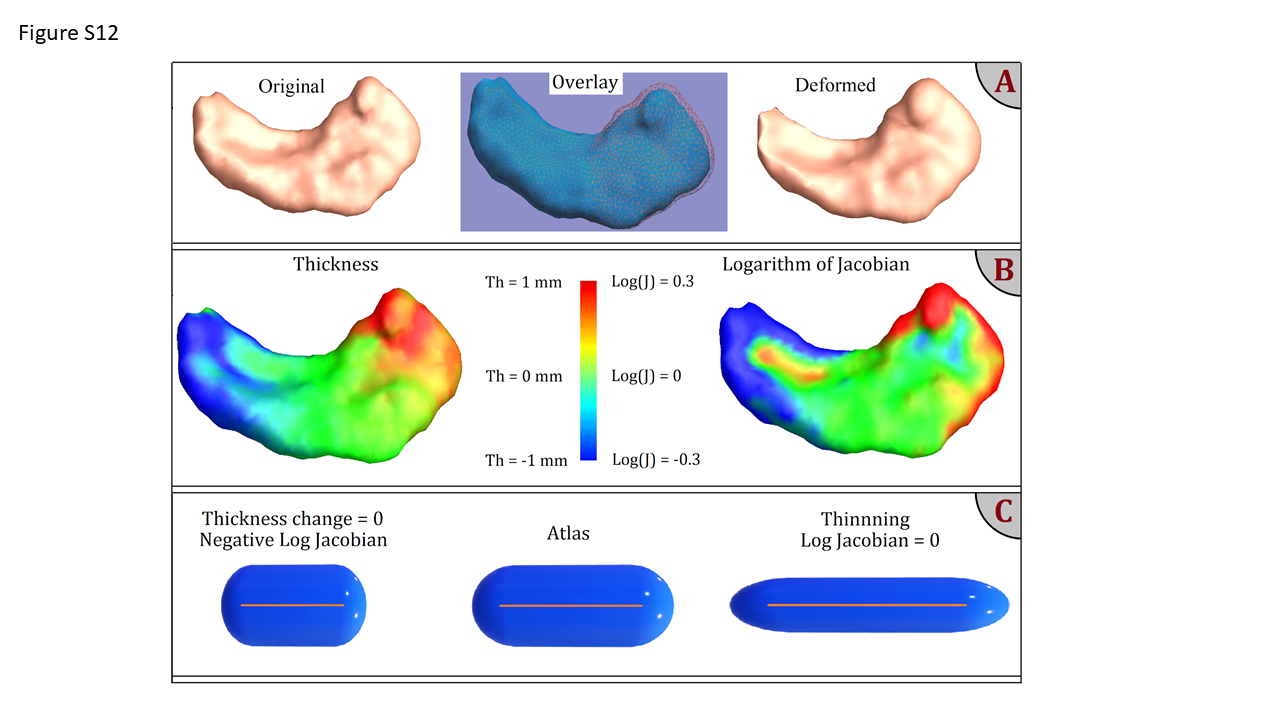


**Additional Acknowledgments**

The CIAM study (FMH - PI) was supported by the University Research Committee, University of Cape Town and the National Research Foundation, South Africa.

The Dublin study was supported by grant funding from the Irish Health Research Board (grant number HRA_POR/2012/54) and Science Foundation Ireland (grant numbers 12/IP/1359 and 08/IN.1/B1916).

The FBIRN study was supported by the National Center for Research Resources at the National Institutes of Health (grant numbers: NIH 1 U24 RR021992 (Function Biomedical Informatics Research Network) and NIH 1 U24 RR025736-01 (Biomedical Informatics Research Network Coordinating Center; http://www.birncommunity.org). The UCISZ study was supported by the National Institutes of Mental Health grant number R21MH097196 to TGMvE. FBIRN and UCISZ data were processed by the UCI High Performance Computing cluster supported by Joseph Farran, Harry Mangalam, and Adam Brenner and the National Center for Research Resources and the National Center for Advancing Translational Sciences, National Institutes of Health, through Grant UL1 TR000153. FBIRN thanks Mrs. Liv McMillan for overall study coordination.

The FIDMAG study was supported by Miguel Servet Research Contract MS14/00041 and Research Project PI14/00292 from the Plan Nacional de I+D+i 2013–2016, the Instituto de Salud Carlos III-Subdirección General de Evaluación y Fomento de la Investigación and the European Regional Development Fund (FEDER), Juan de la Cierva-formación contract (FJCI-2015-25278), and Cibersam.

The Galway study was supported by grant funding from the Health Research Board (grant number HRA_POR/2011/100) and the Wellcome Trust (grant number 072894/2/03/Z).

The Hubin study was supported by the Swedish Research Council (grant numbers K2009-62X-15077-06-3 and K2012-61X-15077-09-3), the Karolinska Institutet and the Knut and Alice Wallenberg Foundation.

The KaSP study was supported by grants from the Swedish Medical Research Council (SE: 2009-7053; 2013-2838; SC: 523-2014-3467), the Swedish Brain Foundation, Åhlén-siftelsen, Svenska Läkaresällskapet, Petrus och Augusta Hedlunds Stiftelse, Torsten Söderbergs Stiftelse, the AstraZeneca-Karolinska Institutet Joint Research Program in Translational Science, Söderbergs Königska Stiftelse, Professor Bror Gadelius Minne, Knut och Alice Wallenbergs stiftelse, Stockholm County Council (ALF and PPG), KID-funding from the Karolinska Institute.

The MCIC study was supported by the National Institutes of Health (NIH/NCRR P41RR14075 and R01EB005846 (to Vince D. Calhoun)), the Department of Energy (DE-FG02-99ER62764), the Mind Research Network, the Morphometry BIRN (1U24, RR021382A), the Function BIRN (U24RR021992-01, NIH.NCRR MO1 RR025758-01, NIMH 1RC1MH089257 to Vince D. Calhoun), the Deutsche Forschungsgemeinschaft (research fellowship to Stefan Ehrlich), and a NARSAD Young Investigator Award (to Stefan Ehrlich).

The NU study was supported by NIH grants P50 MH071616, R01 MH056584, R01 MH084803 (Wang PI), T32 NS047987 (Cobia PI), U01 MH097435 (Wang, Turner, Ambite, Potkin PIs), R01 EB020062 (Miller, Paulsen, Mostfosky, Wang PIs), NSF 1636893 (Pestilli, Wang, Saykin, Sporns PIs), NSF 1734853 (Pestilli, Garyfallidis, Henschel, Wang, Dinov PIs).

The Olin study was supported by R37MH43375 and R01MH074797.

The PAFIP study was supported by Instituto de Salud Carlos III, FIS 00/3095, 01/3129, PI020499, PI060507, PI10/00183, the SENY Fundació Research Grant CI 2005‐0308007, and the Fundación Marqués de Valdecilla API07/011.

The TOP study was supported by the Research Council of Norway (#213837, #217776, #223273), the South-East Norway Health Authority (2013-123), and the KG Jebsen Foundation.

The UPenn study was supported by National Institute of Mental Health grants MH064045, MH 60722, MH019112, and MH085096 (DHW). Theodore D. Satterthwaite was supported by MH098130 and by the Marc Rapport Family through NARSAD.

The SLF Rome study was supported by the Italian Ministry of Health grant RC-12-13-14-15-16-17-18/A.

The NARSAD and Wellcome studies were supported by grants from FAPESP-Brazil (#2009/14891-9, 2010/18672-7, 2012/23796-2 & 2013/03905-4), CNPq-Brazil (#478466/2009 & 480370/2009), the Wellcome Trust (UK) and the Brain & Behavior Research Foundation (2010 NARSAD Independent Investigator Award granted to Geraldo F. Busatto).

The Queensland Twin IMaging (QTIM) study was supported by the National Institutes of Health (R01 HD HD050735, 1U54EB020403-01, subaward no. 56929223) and the National Health and Medical Research Council (1009064, 496682).

Research reported in this publication was also supported by the following VA grants (VA I01 CX000497 and Senior Research Career Scientist to JMF) and National Institutes of Health grants: U54 EB020403 to PMT, R01 MH116147, U24 RR21992, R21 MH097196, and TR000153 to TGMvE, S10 OD023696 and R01EB015611 to PK, T32 AG058507 and 5T32 MH073526 to CRKC, R01 MH117601 to NJ, R01 DA053028 to LW and JT. The content is solely the responsibility of the authors and does not necessarily represent the official views of the funding agencies.

**References for supplemental materials**

Borenstein, M., Hedges, L. V., Higgins, J. P., & Rothstein, H. R. (2011). *Introduction to Meta-Analysis*: John Wiley & Sons.

Cooper, H., Hedges, L. V., & Valentine, J. C. (Eds.). (2009). *The Handbook of Research Synthesis and Meta-Analysis* (2nd ed.). New York, NY: Russell Sage Foundation.

Harville, D. A. (1977). Maximum Likelihood Approaches to Variance Component Estimation and to Related Problems. *Journal of the American Statistical Association, 72*(358), 320-338. doi:10.2307/2286796

Higgins, J. P., Thompson, S. G., Deeks, J. J., & Altman, D. G. (2003). Measuring inconsistency in meta-analyses. *BMJ, 327*(7414), 557-560. doi:10.1136/bmj.327.7414.557

Viechtbauer, W. (2010). Conducting Meta-Analyses in R with the metafor Package. *Journal of Statistical Software; Vol 1, Issue 3 (2010)*.
